# Design of functionalised circular tandem repeat proteins with longer repeat topologies and enhanced subunit contact surfaces

**DOI:** 10.1038/s42003-021-02766-y

**Published:** 2021-10-29

**Authors:** Jazmine P. Hallinan, Lindsey A. Doyle, Betty W. Shen, Mesfin M. Gewe, Brittany Takushi, Madison A. Kennedy, Della Friend, James M. Roberts, Philip Bradley, Barry L. Stoddard

**Affiliations:** 1 Division of Basic Sciences, Fred Hutchinson Cancer Research Center, 1100 Fairview Ave. North, Seattle, WA 98109 USA; 2 Lumen Bioscience Inc., 1441 North 34th Street, Seattle, WA 98103 USA; 3 Division of Public Health Sciences and Program in Computational Biology, Fred Hutchinson Cancer Research Center, 1100 Fairview Ave. N., Seattle, WA 98009 USA

**Keywords:** Proteins, Nanobiotechnology, X-ray crystallography

## Abstract

Circular tandem repeat proteins (‘cTRPs’) are de novo designed protein scaffolds (in this and prior studies, based on antiparallel two-helix bundles) that contain repeated protein sequences and structural motifs and form closed circular structures. They can display significant stability and solubility, a wide range of sizes, and are useful as protein display particles for biotechnology applications. However, cTRPs also demonstrate inefficient self-assembly from smaller subunits. In this study, we describe a new generation of cTRPs, with longer repeats and increased interaction surfaces, which enhanced the self-assembly of two significantly different sizes of homotrimeric constructs. Finally, we demonstrated functionalization of these constructs with (1) a hexameric array of peptide-binding SH2 domains, and (2) a trimeric array of anti-SARS CoV-2 VHH domains. The latter proved capable of sub-nanomolar binding affinities towards the viral receptor binding domain and potent viral neutralization function.

## Abstract (web summary)

Jazmine Hallinan et al. report the development of a new generation of circular tandem repeat proteins with enhanced self-assembly. Functionalisation of these constructs with SARS CoV-2 VHH domains resulted in sub-nanomolar binding affinity to the viral receptor binding domain.

## Introduction

Protein engineering has enabled the creation of novel protein folds and assemblages, as well as the modification of existing protein molecules^1^. The discipline of protein engineering has been greatly facilitated by at least three significant recent advances: the development of accurate computational algorithms for de novo structural design, an enhanced ability to construct and screen large protein libraries, and increasingly efficient and affordable deep sequencing approaches. Whether they are created via de novo computational design or laboratory selections (or a combination of those two approaches), engineered proteins are now being used for a wide variety of biotechnology applications, including those that can benefit from new ligand binding proteins^2–4^, novel immunological regulators^5^ or targeted protein therapeutics^6,7^.

Tandem repeat proteins (‘TRPs’) contain modular units of repeated protein sequence and structure, that can be composed of a variety of structural motifs including α-helical bundles, β-sheets, or mixed topologies^8–11^. They are particularly amenable to de novo design via purely computational approaches, due to their highly modular architectures^8,9^. Multiple types of computationally designed tandem repeat proteins (using either naturally existing or computationally designed motifs and topologies) have been described that form either extended linear shapes (in which the N- and C-termini are at opposite ends of the protein chain)^12^ or closed circular shapes (in which the N- and C-termini contact with one another and thereby complete a closed protein ring)^5,13,14^. Engineered TRPs display many of the same properties as naturally evolved repeat proteins, including relatively few constraints on their length and size, significant flexibility that allows them to sample a wide variety of curvature and shapes, and a propensity to exhibit high thermostability and solubility.

We have previously described the creation of an array of circular tandem repeat proteins (‘cTRPs’) that are constructed from repeated two-helix bundles and that display a wide range of sizes and symmetries^5,13^. The largest of these constructs was used to create symmetric protein nanoparticles that can incorporate a variety of functional protein via covalent attachment around their periphery^5^. cTRPs that display multiple binding domains can display significant avidity effects (due to multimeric display of those domains around the molecular surface) and have been used to create novel formulations of immunological stimulatory and signaling molecules that can be used for biotechnology applications such as therapeutic T-cell manufacture.

While these cTRP constructs have many favorable properties, we have previously noted that their ability to self-assemble from smaller subunits is compromised by relatively limited contacts and small surface areas that are involved in packing between repeats and subunits. For example, a designed cTRP that was intended to form a homotrimer (corresponding to noncovalently assembled cTRP composed of three subunits that contain three repeats each) was instead found to inefficiently form a tetramer, indicating that the structural contacts between repeats and subunits was inadequate to enforce a desired stoichiometric assemblage pattern^13^. Similarly, a series of cTRP subunits designed to self-assemble into a 24-repeat assemblage failed to assemble altogether, indicating that the structural contacts between repeats and subunits were inadequate to drive efficient assembly, a deficiency that was addressed through the incorporation of disulfide ‘staples’ across each subunit interface^5^.

Based on those results, we hypothesized that a new generation of computationally designed cTRP proteins with increased repeat size (corresponding to longer secondary structural elements that increase both particle thickness and the number of buried contacts between repeats and protein subunits) would improve both the energetics and the stoichiometric control of self-association. In this study, we describe the de novo design of thick cTRPs (‘tcTRPs’) with repeats composed of longer helical bundles that form a new underlying repeat topology and demonstrate their solution and assembly behaviors, their structural features via X-ray crystallography and CryoEM, and their ability to be functionalized with additional folded protein domains.

## Results

### Design, expression, and solution behavior of tcTRP9 and tcTRP24

We previously developed an approach to geometry-guided repeat protein design that was described in^13,15,16^ and is implemented in the Rosetta molecular modeling package^17,18^. Key features include symmetry of backbone and side-chain conformations extended across all repeats (allowing computational complexity to scale with repeat length rather than protein length); a pseudo-energy term that favors the desired inter-repeat geometry; clustering and resampling stages that allow intensified exploration of promising topologies; and an in silico validation step that assesses sequence-structure compatibility by attempting to re-predict the designed structure given only the designed sequence. When applying this approach to the design of closed tandem repeat proteins with increased thickness (‘tcTRPs’ or ‘thick(er) circular tandem repeat proteins’), we identified designs with repeats corresponding to right-handed helical bundles as displaying favorable predicted folding energetics for their designed backbone topologies, and well-formed energy funnels when subjecting their sequences to unbiased fold predictions in *Rosetta*. We ultimately selected a small number of individual designs corresponding to two different tcTRP sizes and symmetries (containing 9 repeats and 24 repeats, respectively) to examine and validate using biophysical and structural approaches.

**Fig. 1:**
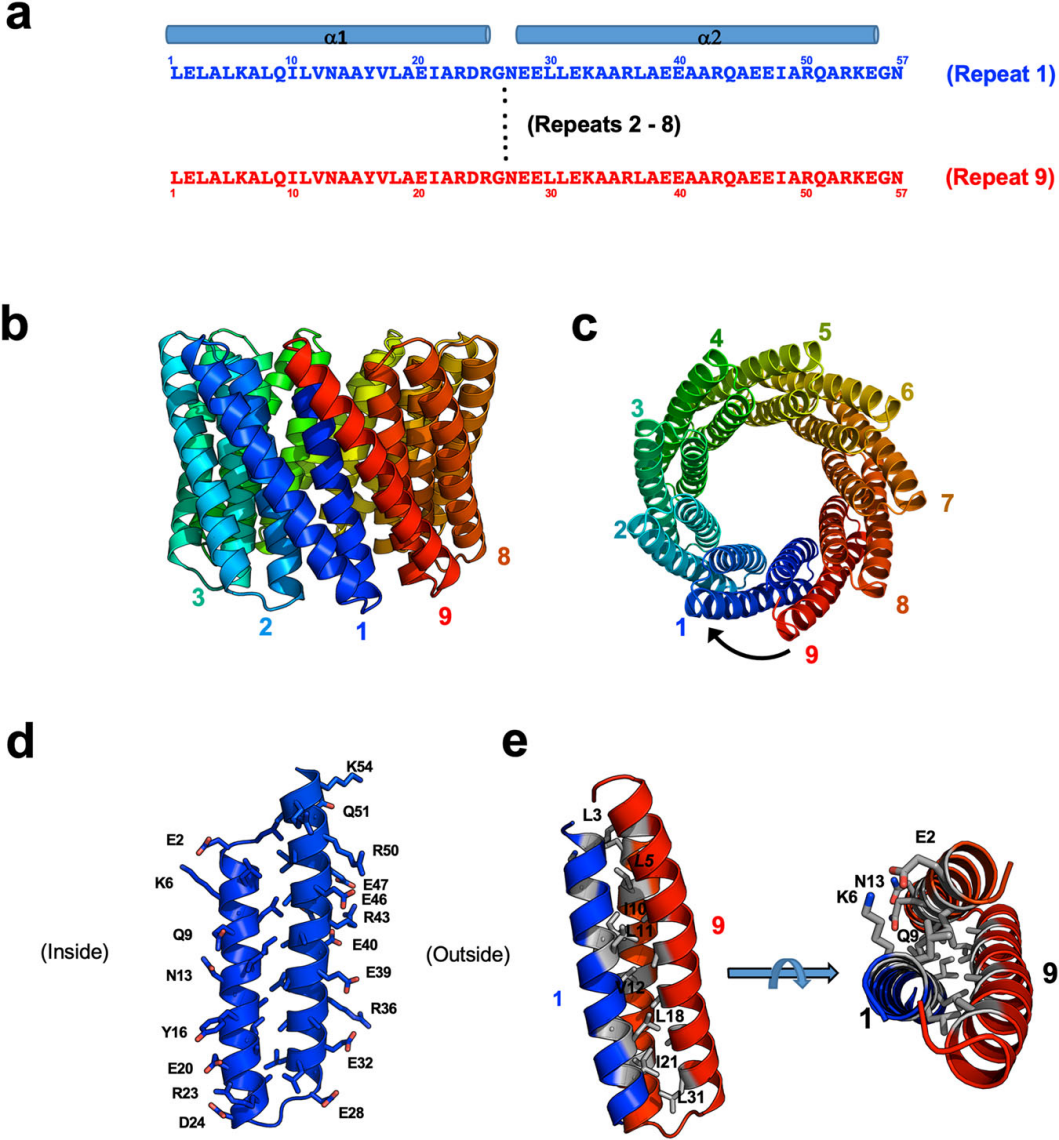
Design of tcTRP9. **a** Sequence and secondary structure of individual repeats, which each consist of an anti-parallel two-helix bundle connected by short turns composed of ‘GN’ sequences. **b**, **c** Nine consecutive repeats (colored blue at the N-terminal repeat and progressing to red at the C-terminal repeat in all figure panels) form a closed cylindrical structure, with an exterior diameter of ~60 Å and an interior pore diameter of ~15 Å. **d** The structural composition of the two-helix bundle corresponding to each designed repeat places mostly charged side chains (largely glutamic acid and arginine residues) on the exterior of the particle, and a network of charged and hydrophilic residues (along with one aromatic tyrosine) on its interior surface. The interface between the two helices is largely composed of a series of leucine and alanine residues (not labeled). **e** The interface between individual repeats corresponds to a series of beta-branched (Ile and Val) and gamma-branched (Leu) hydrophobic residues that form a hydrophobic core between helices, flanked by hydrophilic residues extending towards the interior and exterior of the tcTRP particles.

**Fig. 2:**
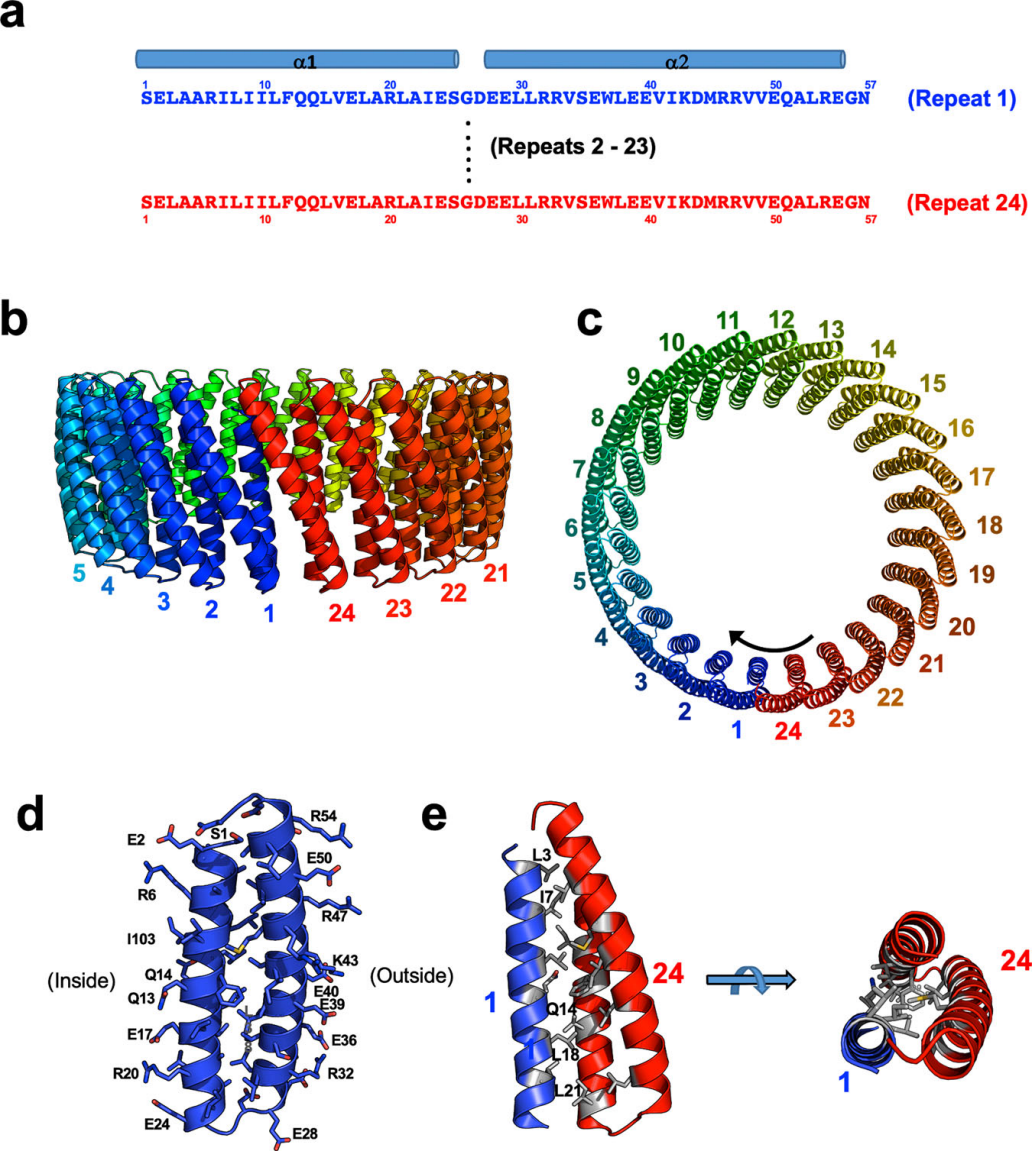
Design of tcTRP24. **a** Sequence and secondary structure of individual repeats, which each consist of an anti-parallel two-helix bundle connected by short turns composed of ‘GD’ sequences. **b**, **c** Twenty-four consecutive repeats (colored blue at the N-terminal repeat and progressing to red at the C-terminal repeat in all figure panels) form a closed cylindrical structure, with an exterior diameter of ~100 Å and an interior pore diameter of ~60 Å. **d** The structural composition of the two-helix bundle corresponding to each designed repeat is chemically similar to that of the tcTRP9 design (Fig. 1), again placing mostly charged glutamic acid and arginine residues on the exterior of the particle, and a network of charged and hydrophilic residues on its interior surface. **e** The interface between individual repeats.

The smaller of the two designed tcTRPs (‘tcTRP9’; Fig. 1) was initially expressed and purified both as a monomeric construct containing 513 residues (MW = 56.6 kDa) composed of nine equivalent repeats of 57 residues each, and as a smaller protein chain (‘tcTRP9_3_’) containing three repeats (172 residues; MW = 18.9 kDa) that was intended to assemble into a trimeric tcTRP particle with the same architecture and dimensions as its single-chain parent (with an exterior diameter of ~60 Å and an interior pore ~15 Å across). The larger of the designed tcTRPs (‘tcTRP24’; Fig. 2) was generated solely from smaller subunits containing either six repeats (‘tcTRP24_6_’) or eight repeats (tcTRP24_8_’) that were intended to assemble into tetrameric or trimeric tcTRP particles, each with an overall architecture and dimensions corresponding to the original design of a single-chain tcTRP containing 24 repeats in total (with an exterior diameter of ~100 Å and an interior pore ~ 60 Å across).

**Fig. 3:**
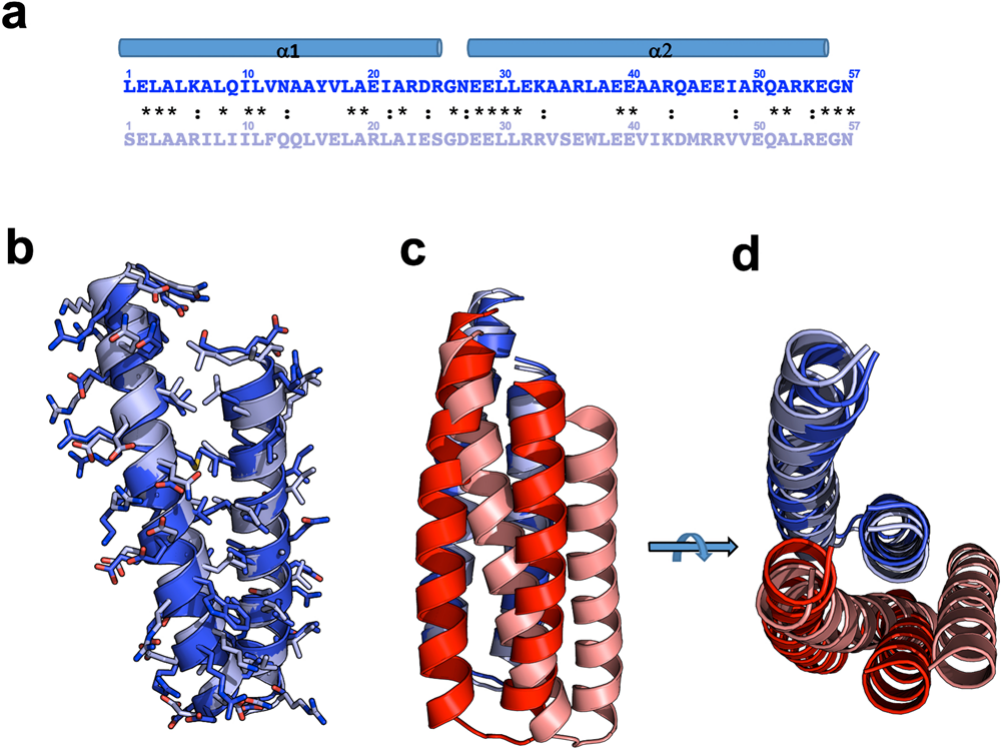
Comparison of designed repeats from tcTRP9 and tcTRP24. Repeats from tcTRP9 and tcTRP24 are shown with darker shades and lighter shades, respectively. **a** Alignment of the sequences of an individual repeats from each of the two designs. Both designs are based on a 57-residue sequence that forms an anti-parallel two-helix bundle. The overall sequence identity (noted by asterisks) is 37%; sequence similarity (including D/E, N/Q, K/R, and I/V pairs) is 53%. Many of the identities between designs are observed at less-constrained surface-exposed positions, but also are found at interfacial positions that contribute to packing between helices and repeats. **b** Superposition of individual repeats from tcTRP9 and tcTRP24 gives an overall rmsd across all alpha carbons of approximately 1.5 Å, indicating that the backbone architecture of individual repeats from the two designs is similar. **c**, **d** Superposition of a single repeat from tcTRP9 and tcTRP24 (dark and light blue, respectively) and extension to the neighboring immediate downstream repeat (dark and light red, respectively) illustrates that the curvature and diameters between the two constructs are facilitated by altered packing angles between sequential repeats (resulting in an alpha-carbon rmsd value between the downstream repeats of over 4 Å).

The differences in the design of repeat sequences, structures, and packing between the tcTRP9 and tcTRP24 models are illustrated in Fig. 3. The sequences of individual repeats from the two designs are similar to one another: each corresponds to a repeated 57 residue sequence that forms an anti-parallel two-helix bundle, with overall 37% sequence identity (50% similarity). The identities and similarities at individual residues between repeats in the two designed proteins are found both at surface-exposed positions and at interfacial positions that contribute to packing within individual repeats. The overall rmsd for all alpha carbons within comparable repeats in the two constructs is approximately 1.5 Å (dark and light blue, respectively in Fig. 3b–d), indicating that the backbone architecture of repeats from the two designs is quite similar. However, superposition of a single repeat from tcTRP9 and tcTRP24 and examination of the deviation in the positions of adjacent downstream repeats (red and salmon respectively in Fig. 3c, d) illustrates that the differences in the size and curvature between the two constructs are facilitated by altered packing angles between sequential repeats (resulting in differences in the positions of the immediately adjacent, downstream repeats of over 4 Å rmsd).

### Solution behavior and crystal structures of tcTRP9 and tcTRP9_3_

To validate the accuracy of the tcTRP9 particle (harboring nine repeats), the recombinant protein was overexpressed and purified, its apparent molecular mass analyzed by size exclusion chromatography, its thermal stability verified by circular dichroism (CD) spectroscopy and its crystal structure determined. The protein was overexpressed and purified to homogeneity (Supplementary Fig. S1a) with a yield of approximately 4 mg per liter of LB culture media. The protein eluted from an SEC column in a single relatively uniform peak at a retention volume corresponding to the mass of the protein (56 kDa) (Supplementary Fig. S1b, left). CD spectra collected at 22° and 95° were nearly superimposable and indicated the presence and retention of the helical secondary structure after heating (Supplementary Fig. S1b, right). The thermal stability of these constructs was comparable to previously designed cTRP constructs^5,13^.

**Fig. 4:**
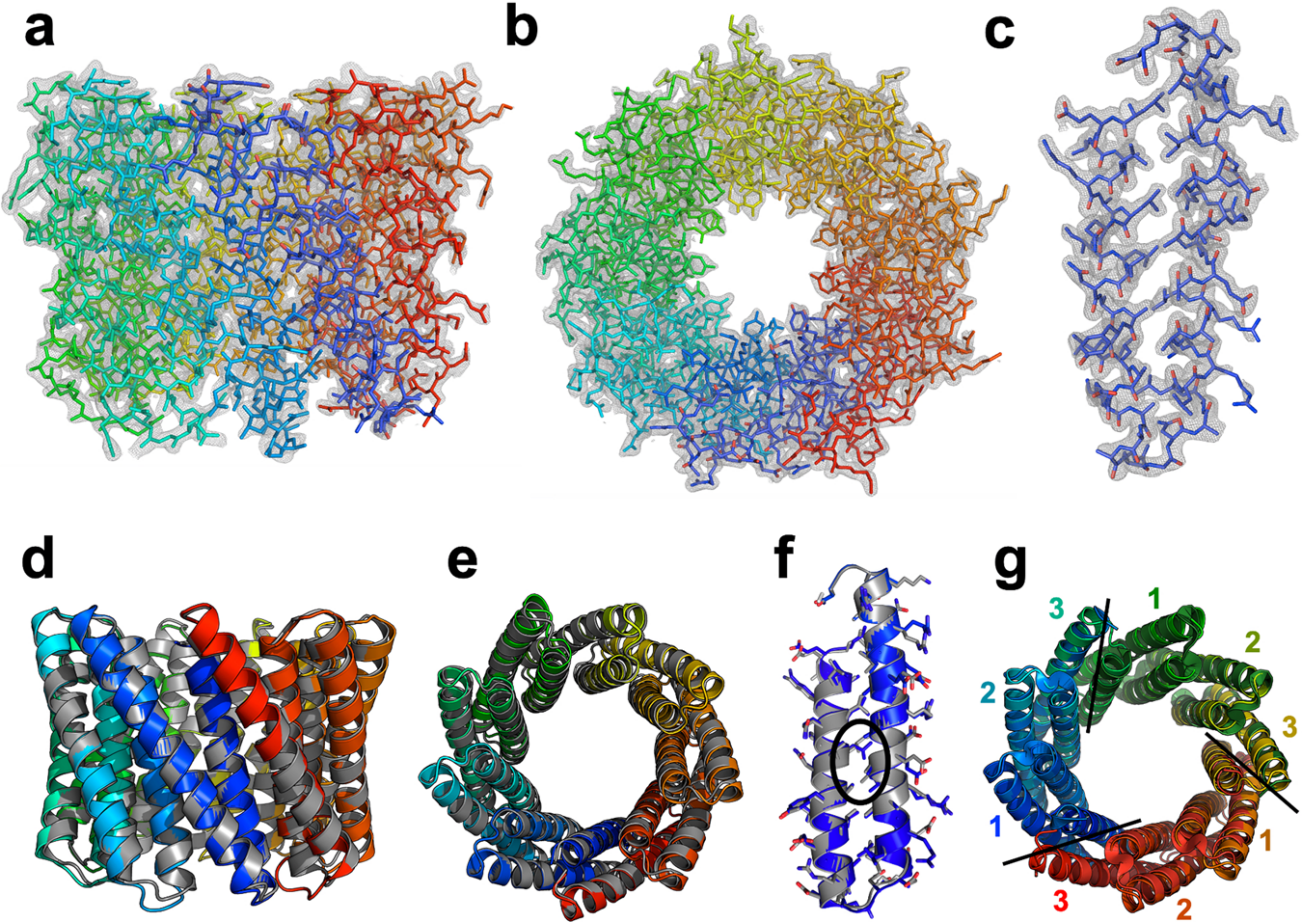
Crystal structures of tcTRP9 and tcTRP9_3_. **a**–**c** Refined models and corresponding 2Fo-Fc electron density map for full-length monomeric tcTRP9. **c** Illustrates the map and refined model for a single repeat within the construct. The structure was determined at 2.1 Å resolution. **d**–**f** Superposition of the designed model of tcTRP9 (gray) and the experimental crystallographic structure of the same construct (rainbow spectrum; N-terminal repeat = blue, C-terminal repeat = red). The alpha-carbon rmsd between the design and the refined crystallographic coordinates is 0.5 Å. The circled residues in **f** (which shows the side chains for repeat #1 from the design and from the crystal structure) are L11 and L37, which pack against one another in both models, but display opposing rotameric conformations that each promote their interaction in the interhelical interface of the repeat. **g** Superposition of the crystal structure of the monomeric tcTRP9 construct (from the earlier panels) and the crystal structure tcTRP9_3_ homotrimer (a self-assembling particle-containing three subunits, each of which contains 3 repeats of the same sequence as shown in Fig. 1; the structure was solved at 3.2 Å resolution). The structure of the latter construct confirms its homotrimeric assembly (as previously indicated by size exclusion chromatography analysis) and alpha-carbon rmsd between superimposed monomer and homotrimer of 0.5 Å. The repeat numbering (1−3, repeated three times) and lines illustrate the subunit boundaries and locations for the homotrimeric tcTRP9_3_ construct.

The crystal structure of the tcTRP9 particle (Table 1 and Fig. 4a–c), determined to 2.1 Å resolution, validated the accuracy of the design. Superposition of the design model and the experimental crystal structure (Fig. 4d–f) produced an rmsd value for all alpha-carbons of ~0.5 Å, and close agreement between side-chain rotamers within the inter- and intrahelical repeats and interaction surfaces. The only positions where the side-chain rotameric conformation significantly differs between the design and the structure is for a pair of leucine residues (L11 and L37), which interact with one another through van der Waals interactions between their side chains in both models.

**Table 1:** Crystallographic data collection and refinement statistics.

	tcTRP9 (monomer)	tcTRP9_3_ (homotrimer)
*Data collection*		
Space group	P2_1_	P2_1_
Cell dimensions		
*a*, *b*, *c* (Å)	52.5, 85.5, 56.1	57.6, 78.9, 112.7
*α*, *β*, *γ* (°)	90, 103.6, 90	90, 93.8, 90
Resolution (Å)	2.1(2.18–2.1)^a^	3.2 (3.31–3.2)^a^
*R*_sym_ or *R*_merge_	0.036 (0.109)	0.221 (0.698)
*I*/σ*I*	40.4 (14.0)	8.5 (3.4)
Completeness (%)	99.5 (95.7)	88.0 (90.0)
Redundancy	7.3 (5.9)	5.5 (5.5)
*Refinement*		
Resolution (Å)	2.1	3.2
No. reflections	28299	16744
*R*_work_/*R*_free_	0.1701/0.2277	0.2480/0.3302
No. atoms		
Protein	3870	7053
Ligand/ion	0	0
Water	308	12
*B*-factors		
Protein	28.65	30.27
Ligand/ion	34.01	20.20
Water		
R.m.s. deviations		
Bond lengths (Å)	0.007	0.003
Bond angles (°)	0.75	0.72

^a^1 crystal for each data set.

Because the original (thinner) “cTRP9” construct (also harboring nine repeats; Supplementary Fig. S2a) was previously observed not to properly self-assemble from smaller subunits^13^ (inefficiently forming an unexpected tetramer at high protein concentrations, instead of the intended trimer), we tested and compare the ability of the thicker “tcTRP9” scaffold (Supplementary Fig. S2b) to self-assemble from smaller subunits. To accomplish this, we generated and purified a construct that again contained only three repeats per subunit (termed ‘tcTRP9_3_’). This construct was also expressed and purified at relatively high levels (Supplementary Fig. S1a; yield ~5 mg per liter of culture) and eluted from a final SEC column at a volume corresponding to the same approximate mass as the full-length protein (Supplementary Fig. S1b, left), indicating probable assembly into a higher-order trimeric species with 9 repeats total. The purified construct, also like the full-length protein, did not display significant unfolding behavior at 95° in a CD spectrum (Supplementary Fig. S1b, right). The purified construct from the final SEC column was crystallized and its structure was determined at 3.2 Å resolution (Table 1). This analysis confirmed a homotrimeric assembly that closely mirrored the dimensions, topology, and side-chain packing interactions found in the full-length tcTRP9 designed protein (Fig. 4g).

### Generation and analysis of tcTRP24_8_

Having demonstrated the accuracy and behavior of a designed thick(er) cTRP (tcTRP) harboring nine repeats and a relatively acute radius of curvature between sequential repeats, we next decided to generate and examine a similar tcTRP harboring 24 repeats, corresponding to a considerably larger internal and external diameter and a shallower curvature between consecutive repeats. Because the large size of these constructs (and the corresponding large number of repeats within their designed structures) precluded generating monomeric, single-chain constructs of 24 linear repeats, we decided to immediately test our designs using smaller subunits with fewer number of repeats that were again intended to self-assemble to form full-sized tcTRP toroidal particles.

Designs corresponding to subunits containing either 6 (tcTRP24_6_) or 8 (tcTRP24_8_) repeats, that were respectively intended to assemble into a full-sized particle with 24 repeats via tetramerization or trimerization (Supplementary Fig. S3a, left) were found to both express at high levels (Supplementary Fig. S3a, right). The tcTRP24_8_ construct was slightly better behaved in subsequent purification attempts and was therefore used for further experiments. The protein was purified to relative homogeneity using a three-column protocol (“Methods” and Supplementary Fig. S3b).

**Fig. 5:**
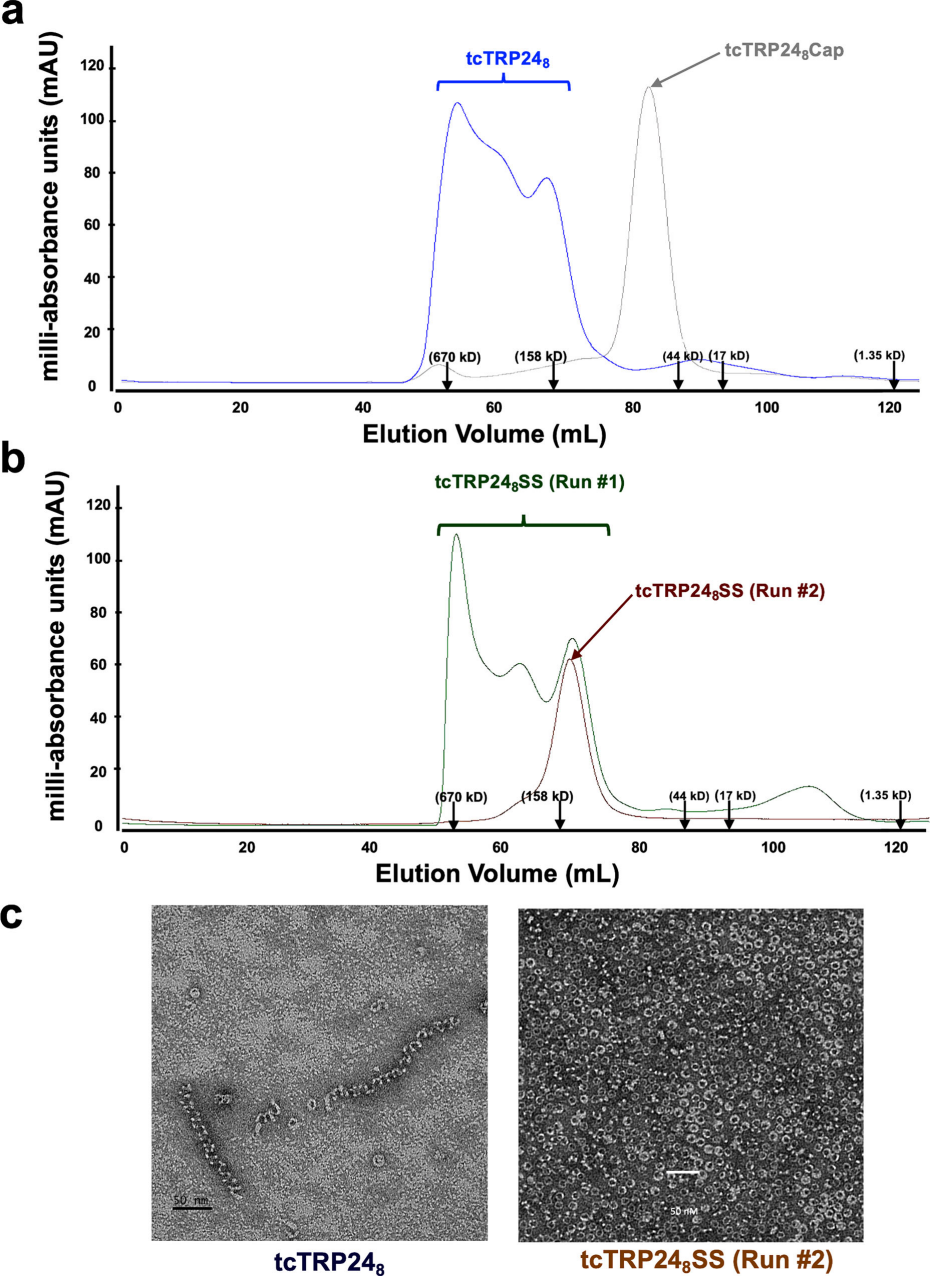
Assembly behavior of tcTRP24_8_ and tcTRP24_8_SS. **a** Size-exclusion chromatographic (SEC) elution profiles of the original ‘tcTRP24_8_’ homotrimeric construct (blue), and the ‘tcTRP24_8_Cap’ construct (gray), which harbors a pair of disrupting mutations at the subunit interface that prevents self-association. The original construct displays a wide range of apparent masses, with the final peak corresponding to the approximately expected elution and mass for the desired homotrimeric species. **b** SEC elution profiles of the ‘tcTRP24_8_SS’ homotrimeric construct directly from the initial purification out of bacterial cells (‘Run#1’, green), and after collecting, concentrating, and re-running the tail of the original, properly sized fractions (‘Run#2’, brown). The original prep (Run#1) still forms a distribution of masses after purification, albeit with the earliest and latest eluting peaks increased in relative percentage of material. However, collection of the late-arriving fractions corresponding to the predicted mass of the intended homotrimer and a second run over the same column now results in a single uniform peak corresponding to the intended mass of the designed homotrimeric construct. **c** Negative stain EM image of tcTRP24_8_ (left) and of tcTRP24_8_SS (‘Run #2’; right). The original construct (lacking disulfide staples; left panel) displays a mixture of correctly sized trimeric particles (small rings on the micrograph) and long extended fibers. The resized stapled construct (right panel) displays a uniform distribution of tcTRP particles. Subsequent CryoEM analyses of this construct (Supplementary Figs. S4 and 6) verify the symmetry, size, and repeat copy number of the construct.

The tcTRP24_8_ construct eluted from the final SEC column over a wide range of elution volumes (Fig. 5a), corresponding to a heterogeneous population of sizes ranging from greater than 670 kDa to a final peak corresponding to a mass slightly greater than 158 kDa (near the expected mass of individual tcTRP particles composed of three subunits with a total of 24 repeats). Fractions from the SEC elution were subsequently subjected to TEM imaging of negative stained specimen (Fig. 5c, left panel). The resulting images contained mixtures of individual open (‘c’-shaped) and closed (circular) toroidal particles with the diameter of the latter corresponding to the expected dimension of the designed protein. Those particles were interspersed with fibrous assemblages that appeared to correspond to elongated chains of protein subunits, forming spiral assemblages of variable lengths. The thickness of the fibers was similar to the diameter of the neighboring rings.

Based on those images, we reasoned that the tcTRP24_8_ design might be forming a mixture of desired circular particles, along with partially closed assemblages with an exposed interface surface at their termini that might promote the incorporation of additional protein subunits (leading to a mixture of unassembled subunits, closed toroidal particles, and the growth of fibers until free protein was exhausted). We therefore attempted to promote the closed toroidal form of this construct by incorporating a pair of cysteine residues at neighboring positions across each subunit interface so that a disulfide staple might lock the protein into the desired closed topology, as described previously^5,19^. Based on the tcTRP24_8_ design, an alanine at position 5 and an isoleucine at position 7 (respectively located within the N- and C-terminal repeats of each subunit) were mutated to cysteine residues, with the intention of installing potential disulfide bonds within each interface of the intended tcTRP24_8_ trimeric particle (‘tcTRP24_8_SS’). This construct was again well-expressed in E. coli and purified using the same three-column protocol including a terminal size exclusion chromatography (SEC) step. Like the original tcTCRP24_8_ construct, the protein again eluted over a range of volume and corresponding masses extending from approximately 150 to > 670 kDa. However, the peak corresponding to the mass of the desired trimer appeared to be greater in relative height (Fig. 5b, ‘tcTRP24_8_SS Run #1’).

Reasoning that the designed disulfide bond might only form after cell lysis and exposure to a non-reducing environment (and during that time, would compete with misfolding and fiber formation), we isolated the slowest eluting half of the final peak (corresponding to the expected elution of a correctly formed trimeric particle), concentrated the protein, and subjected it to a second size exclusion step and analysis (Fig. 5b, ‘tcTRP24_8_SS Run #2’). In that second run, the protein now ran as a single uniform peak at a volume and predicted mass corresponding to the desired trimeric assemblage. To further test that conclusio6, fractions of tcTCRP24_8_SS from this second SEC elution were again subjected to transmission electron microscopy using negative stained specimens (Fig. 5c, right panel) and found to correspond to individual toroidal particles with dimensions close to the computational design of a 24-repeat toroid.

A negative control construct (‘tcTRP24_8_Cap’), containing bulky side-chain substitutions in the protein interface that were intended to block self-assembly through steric clashes, was observed to elute at a later volume and smaller corresponding mass, further validating the assembly of the tcTRP24_8_ and tcTRP24_8_SS constructs (Fig. 5a).

**Fig. 6:**
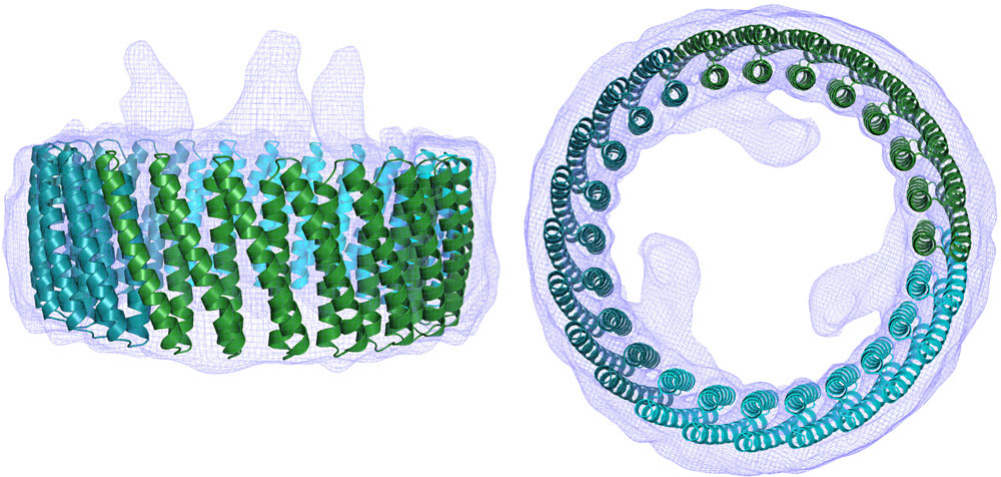
CryoEM single-particle reconstruction of tcTRP24_8_SS. Two different views of the electron density map superposed with the original model. The three-strong features extending from equivalent positions around the tcTRP particle correspond to N-terminal poly-histidine affinity tags and linkers extending from the N-terminus of each protein subunit (an unbiased feature that further validated the trimeric assemblage of the particle). Those atoms are clearly disordered and are not modeled into the density.

The structure of the isolated tcTRP24_8_SS construct from the final SEC run was then further examined by CryoEM single particle analysis (Fig. 6, Table 2 and Supplementary Fig. S4). The resulting density map at a resolution of approximately 6.9 Å, at a Gold Standard Fourier Shell Correlation (GSFSC) of 0.143 between the two half maps, clearly indicated the presence of a close circular-shaped particle with two concentric layers of helices lining its periphery. The topology of the map is consistent with the trimeric tcTRP24_8_SS toroidal assembly and is closely superimposed on the original design model (Fig. 6). In the final electron density map, three strong features extending from equivalent positions around the tcTRP particle correspond to N-terminal poly-histidine affinity tags and linkers extending from the N-terminus of each protein subunit (an unbiased feature that further validated the trimeric assemblage of the particle). Those atoms are clearly disordered and are not modeled into the density.

**Table 2:** Cryo-EM data collection, refinement, and validation statistics.

	tcTRP24_8_SS (EMD-24425) (PDB 7RDR)
*Data collection and processing*	
Magnification	38 Kx
Voltage (kV)	200
Electron exposure (e–/Å^2^)	40
Defocus range (μm)	−0.8 to −2.3
Pixel size (Å)	1.16
Symmetry imposed	C3
Initial particle images (no.)	627763
Final particle images (no.)	121426
Map resolution (Å)	6.9
FSC threshold	0.143
Map resolution range (Å)	30–6.9
*Refinement*	
Initial model used (PDB code)	tcTRP24_8_SS
Model resolution (Å)	6.9
FSC threshold	0.143
Model resolution range (Å)	30–6.9
Map sharpening *B* factor (Å^2^)	−418.6
Model composition	
Non-hydrogen atoms	None
Protein residues	1368
Ligands	None
*B* factors (Å^2^)	
Protein	20.0
Ligand	N/A
R.m.s. deviations	
Bond lengths (Å)	0.01
Bond angles (°)	1.2
Validation	
MolProbity score	1.6
Clashscore	1.05
Poor rotamers (%)	0
Ramachandran plot	
Favored (%)	98.32
Allowed (%)	1.68
Disallowed (%)	0

Inspection of the images from the selected 2D class averages of particles used in the reconstruction indicated that the protein assemblage, even with designed disulfide staples in place at the interfaces between protein subunits, displayed significant conformational flexibility or ‘breathing’ that are manifested in slight deviations from circularity and small but significant variations in the diameter of the particles, which severely limited the resolution of the 3D reconstruction.

In the same CryoEM analyses, we also noted that while the vast majority of the 2D class averages of the closed circular tcTRP24_8_SS particles contained 24 repeats as designed, a small fraction of particles (less than 5%) contained more or less than 24 repeats (Supplementary Fig. S4), indicating the presence of ‘*n* +/− 1’ and/or ‘*n* +/− 2’ repeats in a small fraction of the underlying tcTRP subunits. One possible explanation for this observation could be occasional recombination events within the repetitious tcTRP24_8_ coding sequence, leading to the insertion or deletion of individual DNA repeats from the expression vector. Using PCR, we compared the length of the coding sequence inserts in the original expression vector with plamid populations recovered from cells after extended culture growth and expression but did not see any indication of abnormal length inserts accumulating during cell growth and expression. Thus, the basis for the small percentage of tcTRP24_8_ particles harboring more or less than 24 repeats is unclear.

### Generation and analysis of functionalized tcTRPs

Finally, we tested the ability of the tcTRP24_8_ and tcTRP9_3_ constructs to promote the display and function of multiple copies of a functional protein domains, as has been described previously for other engineered protein scaffolds^5,6,20–22^. For this purpose, we conducted experiments using two underlying tcTRP trimeric constructs (tcTRP24_8_ and tcTRP9_3_).

**Fig. 7:**
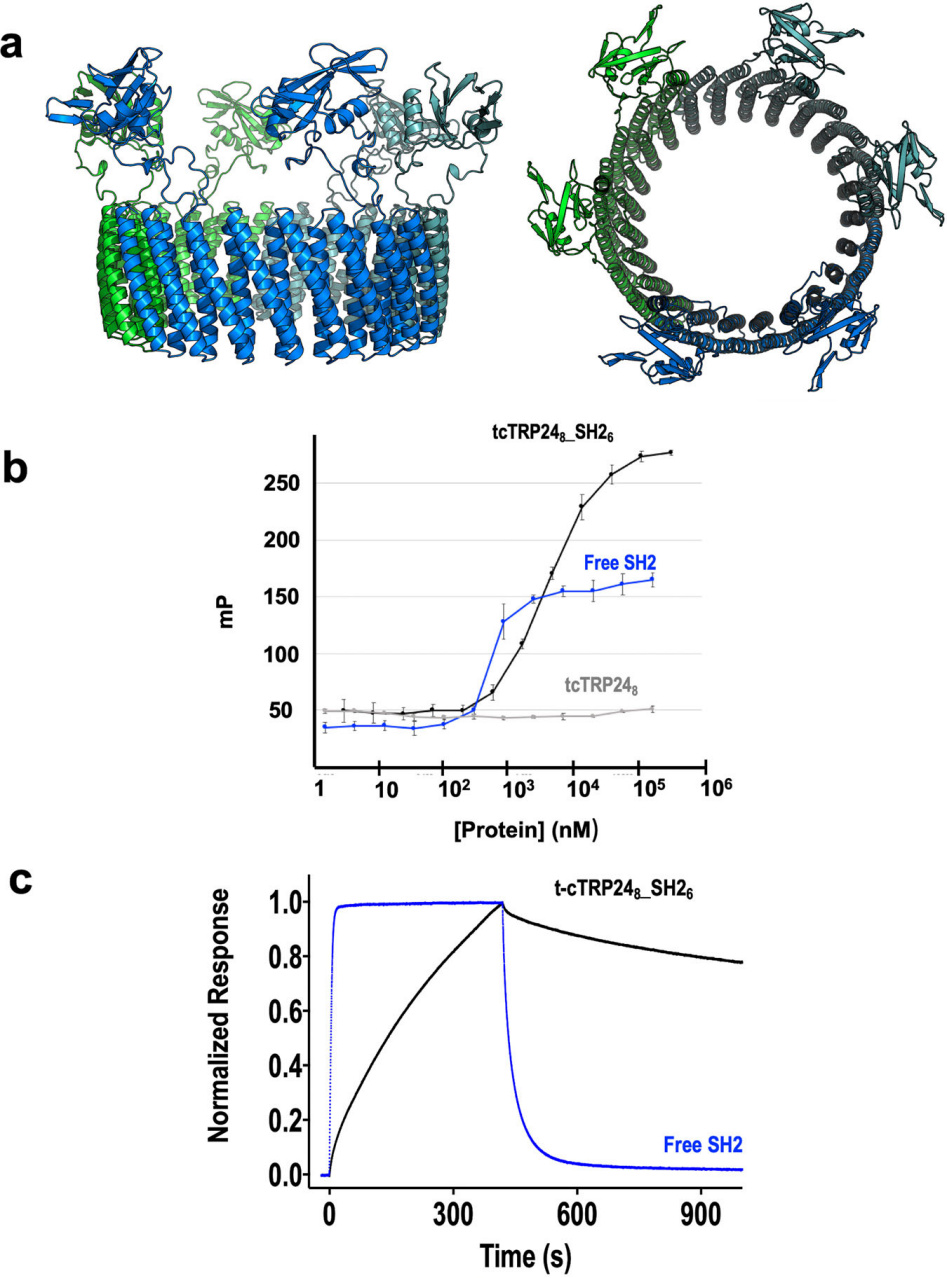
Peptide binding function of tcTRP24_8_SS_SH2_6_ in solution and on a surface. **a** Schematic model of the construct shown in two orientations. Two copies of the SH2 domain are fused into a loop (between repeats 2 and 3, and between repeats 6 and 7) in each of three subunits, resulting in the presence of six domains distributed around the periphery of the construct in a hexagonal arrangement. **b** Binding of fluorescently labeled Tir10 phosphotyrosyl peptide (corresponding to the physiological binding target for the Nck SH2 domain) was measured in solution using fluorescence polarization. Binding activity was measured using isolated SH2 domain (blue), and tcTRP24_8_-SH2_2_ (a tcTRP trimer containing two copies of fused SH2 domains per subunit, i.e., six copies total; black). A control experiment was also conducted using the free ‘naked’ tcTRP24_8_ (gray) that contained no functional protein domains. The protein concentration is normalized to account for the ratio of SH2 domains per molecule (6 SH2 domains per tcTRP_8__SH2_2_ versus 1 per free SH2). Both constructs display saturable binding and an approximate *K*_D_ of 1−3 micromolar. All binding experiments were conducted in triplicate using independent aliquots of each protein. Data shown as mean and standard deviation for *n* = 3 measurements. **c** Binding and dissociation of free SH2 (blue) and cTRP24_8_-SH2_2_ (black) to surface-bound peptide. Surface Plasmon Resonance (‘Biacore’) was used to measure the binding to captured biotinylated phosphotyrosyl peptide as described in Methods.

In the first experiment, we used fluorescence polarization and surface plasmon resonance (SPR) to demonstrate (1) that the peptide-binding activity of an SH2 domain is maintained when fused to a tcTRP scaffold, and (2) that the association and dissociation behavior of the multimeric SH2-tcTRP construct differs from the free SH2 domain. The tcTRP24_8_ construct was functionalized by inserting two copies of a sequence-specific SH2 domain derived from the Nck adapter protein (used previously for a similar purpose with the original, thinner cTRP24 construct)^13^ into each protein subunit. The assembled particle (‘tcTRP24_8_-SH2_6_’) thereby displayed six total copies of the same SH2 domain arranged around the same face of the protein scaffold, with four repeats separating each domain (Fig. 7a). The construct was then assayed for binding to its cognate phosphotyrosyl-containing peptide (EHI*pY*DEVAAD) both free in solution (using fluorescence polarization; Fig. 7b) and on an immobilized surface (using surface plasmon resonance; Fig. 7c).

The solution-based binding function and affinity of free SH2 domains versus tcTRP displayed SH2 domains, measured via fluorescence polarization, were relatively comparable (approximate *K*_D_ values of 1−3 micromolar). The tcTRP24_8_ scaffold alone did not display significant peptide binding. In this experiment, the labeled ligand is not immobilized on a surface, and individual ligand molecules are bound independently (in a completely non-cooperative manner) by each SH2 domain around the periphery of the particle. Therefore, there is no avidity effect to be expected in this experiment, nor is any effect observed.

In contrast, when the peptide binding experiment was carried out using surface plasmon resonance (with the peptide target presented to the protein on the surface of a chip), a significant avidity effect is expected and observed. We observed a greatly reduced dissociation rate (*k*_off_) from the chip, offset by a reduced rate of association (*k*_on_). While we might have expected to see an increased on-rate coupled to a decreased off-rate, a decreased on-rate could instead be caused a combination of (1) greatly increased molecular mass (252 kDa for tcTRP24_8__SH2_6_, versus 12 kD for free SH2) and/or (2) reduced accessibility of the binding domain after tethering it to the tcTRP scaffold.

**Fig. 8:**
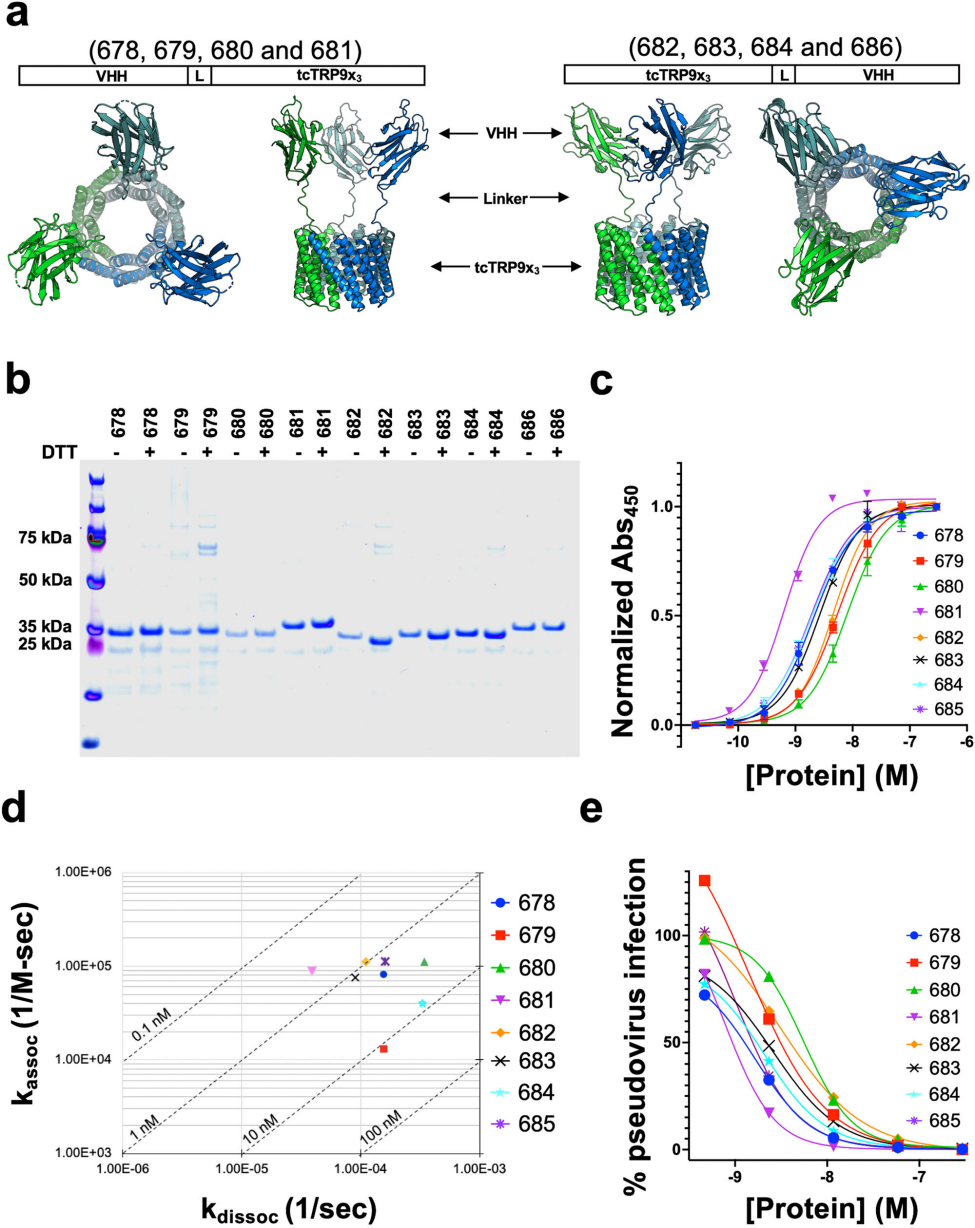
Generation and functional characterization of homotrimeric tcTRP9x_3_-VHH_3_ constructs. **a** Each construct (sequences and locations of inserted VHH domains provided in Supplementary Table S1) corresponds to a fusion at their N- or C-termini with a VHH domain previously shown to bind to the SAR—CoV2 spike protein receptor binding domain^23^. Eight constructs were tested, corresponding to various linkers that fuse the VHH domain to the N- or C-terminal ends of each subunit, in either case generating a trimeric array of VHH domains arranged on one side of the tcTRP9x_3_ scaffold. The ability of each construct to bind the SARS CoV2 spike receptor-binding domain (RBD) was measured using two approaches (Biolayer interferometry and ELISA); the ability of the same constructs to inhibit cell infection was measured using a pseudoviral neutralization assay. **b** Purification of individual constructs from E. coli. Yields ranged from ~15 to ~ 80 mg/L. **c** The relative ability of each construct to bind to the SARS-Cov2 receptor-binding domain (RBD) was measured using an ELISA analysis, leading to EC_50_ values (Supplementary Table S2) ranging from a low of 0.4 nM (construct 681) to 12 nM (construct 680). All binding experiments were conducted in triplicate using independent aliquots of each protein. Data shown as mean and s.d. for *n* = 3 measurements. **d** The relative ability of each construct to bind to the SARS-Cov2 receptor-binding domain (RBD) was measured using Biolayer interferometry (BLI). The rate of association and dissociation are calculated from concentration-dependent responses. The rate of association (*k*_a_) is plotted on the *Y*-axis as inverse molar second (1/MS), and the rate of dissociation (*k*_dis_) is plotted on the *X*-axis as inverse second. Corresponding equilibrium binding constants (*K*_D_) are plotted on a diagonal axis as nano molar concentrations. See also Supplementary Fig. S5. **e** The relative ability of each construct to achieve 50% inhibition of infection of 293T-Ace2 cells using a pseudoviral neutralization assay with SARS-CoV-2 spike pseudotyped lentiviruses was measured. The IC_50_ values (Supplementary Table S2) ranged from 0.78 nM (construct 681) to 5.5 nM (construct 680). All experiments were conducted in triplicate using independent aliquots of each protein. Data shown as the mean and standard deviation for *n* = 3 measurements.

In our second, more comprehensive experiment intended to more rigorously examine the effect of binding domain multimerization, the tcTRP9_3_ construct was functionalized with a previously described camelid ‘nanobody’ (a single chain VHH domain) that was previously raised against the SARS-CoV-1 spike receptor-binding domain (RBD) protein^23^. The VHH contained a point mutation (D61R) that is hypothesized to increase binding to SARS-CoV-2 spike RBD and was fused to the tcTRP domains in several ways, either to each of the three separate N-termini in the trimeric tcTRP or to each of the three separate C-termini (sequences and VHH insertion sites shown in Supplementary Table S1). In both cases, the assembled particles (‘tcTRP9_3_-VHH_3_’) display three separate copies of the antiviral VHH domain in a trimeric organization extending from one face of the construct. For each construct, four different linker sequences were tested in parallel (Fig. 8, Supplementary Tables S1 and S2 and Supplementary Fig. S5).

The tcTRP9_3_-VHH_3_ constructs (Fig. 8a) were expressed and purified from *E. coli* (Supplementary Table S2 and Fig. 8b) and then assayed for binding to the SARS-CoV-2 spike protein RBD (Supplementary Table S2, Fig. 8c, d; Supplementary Fig. S5) using BLI and ELISA methods. The same constructs were then assayed for their ability to block infection of an HEK293 cell line expressing the ACE2 receptor by SARS-CoV-2 pseudoviral neutralization assay (Supplementary Table S2 and Fig. 8e). Protein expression was somewhat variable, depending upon the position (N- or C-terminal) of the VHH domain relative to the tcTRP subunit to which it was fused, and the identity of the linker used to fuse the VHH to the tcTRP repeats. Most of the constructs expressed at high levels corresponding to multiple milligrams of recovered protein per liter of culture, with the constructs corresponding to a C-terminal fusion of the VHH (tcTRP9_3_-linker-VHH-6xHis) displaying more uniform expression and recovery. The trimeric constructs show binding to the SARS-CoV2 RBD and viral neutralization at concentrations lower than or comparable to the reported binding (*K*_D_ ~ 39 nM) and virus neutralization (IC_50_ ~ 4 nM) by the same VHH organized in a dimeric arrangement via fusion to an immunoglobulin Fc domain^23^. The most active construct in our experiments (tcTRP9_3_-VHH681_3_) displayed the lowest, sub-nanomolar values for both binding assays (*K*_D_ and EC_50_ ~ 0.43 and 0.64 nM, respectively, and viral neutralization IC_50_ ~ 0.78 nM).

## Discussion

The study described above expands upon previously published descriptions of de novo designed circular tandem repeat proteins (cTRPs)^5,13^ that were originally inspired by naturally occurring tandem repeat proteins such as TAL (transcription activator-like) effectors^24^, PUF (Pumillio and FBP) proteins^25^, and TPR (tetratricopeptide repeat) proteins^26^. Each of those protein families, which have respectively evolved to bind double-stranded DNA, single-stranded RNA, and peptide targets in a sequence and/or structure-specific manner, are composed of underlying repeats of approximately 34 residues. In carrying out their functions, they display significant plasticity corresponding to interdomain dynamic flexibility, differing amounts of sequence divergence between repeats within individual protein factors, and different ligand-binding mechanisms that generally depend on the identity of residues at canonical positions within each repeat^27^.

The length and helical content of the repeats that were incorporated in our first generation of cTRPs^5,13^ were similar to those found in the naturally occurring repeat proteins listed above, while otherwise exploring geometric constraints and folding topologies (including the requirement of a closed circular architecture) that were unique from those observed in nature or found in previously solved structures. The additional structural requirement for a closed circular architecture imposed the need for heightened accuracy in the design of contacts that lead to precise rotational symmetry, such that the toroidal organization of the folded particle is realized in the correct packing and register between N- and C-terminal repeats at each protein interface. While the final designed scaffolds were well-behaved in many ways (including their expression, solution behavior, thermal stability, and ability to accommodate additional functional protein domains), their ability to assemble from smaller subunits was occasionally observed to be sub-optimal, in terms of how efficiently they assembled^5^ and whether they incorporated the desired number of subunits into the full-sized construct^13^.

In this study, we tested a hypothesis that the expansion of cTRP repeat size and a corresponding increase in the area and residues within the packing interfaces between repeats and subunits might be a route by which the energetics and control of the self-assembly of such constructs could be improved. This appears to have been true, at least in part: the assembly of a trimer containing nine repeats (from subunits containing three repeats each) is enhanced both energetically and sterically relative to the original, comparable design and analysis of a corresponding 9-repeat trimer^13^. Additionally, whereas prior assembly a 24-repeat cTRP construct from smaller subunits was inefficient^5^, the newly designed tcTRP24_8_ construct displayed enhanced assembly (as indicated by size exclusion chromatography analyses that indicated all subunits were incorporated into larger particles). However, assembly of that construct was accompanied by the formation of undesired, higher-order fibrils (Fig. 5c, left) that we believe are likely promoted by ‘off-register’ packing between subunits, leading to a translational component (i.e., a spiral screw-axis) that confounds designed strict rotational symmetry and provides a partially exposed surface at one interface that can act as a nucleation site for subunit addition and continued extension of the protein complex. The introduction of a disulfide staple at the designed interface (which was previously required simply to promote assembly of otherwise disassociated subunits)^5^ was helpful in promoting and trapping desired circularized constructs, which could then be isolated as stable particles for further analyses (Fig. 5c, right). Future studies will examine this behavior, and the computational improvements necessary to prevent this behavior, further.

A wide variety of applications would appear possible for de novo designed protein scaffolds (either tcTRPs, or alternative designed protein particles) that exhibit efficient self-assembly in defined copy number, size, and symmetry. Prior studies with such designed protein scaffolds have demonstrated the creation of high-avidity binding particles^5^, highly active cell stimulatory factors^5^, and epitope display particles for vaccine development^20,22^. In this study, we examine the possibility of functionalizing such designed protein scaffolds for the disruption of biological particles and assemblages, specifically examining how the trimeric assemblage of a previously described camelid nanobody that was selected to bind a viral spike protein receptor-binding domain might improve on such a construct’s viral neutralization properties, relative to a commonly used dimerization motif^23^. It should be possible to expand on this concept for other purposes, such as the degradation of bacterial biofilms (themselves a high copy-number, high avidity target)^28^ or other bioremediation applications. An additional area for investigation might be the use of such self-assembling protein scaffolds for the organization of enzyme domains within a defined pathway, potentially leading to kinetic gains through proximity effects between sequential activities^29,30^.

The comparative examination between various tcTRP trimers augmented with a three-fold arrangement of an anti-SARS CoV-2 VHH domain^23^ demonstrates the overall amenability of the underlying protein scaffold towards variable fusion strategies at the N- or C-terminus of each subunit, as well as when various linker compositions and lengths are employed. At the same time, the fact that one construct (tcTRP9_3_-VHH681_3_) demonstrates consistently superior target binding affinity and biological activity across multiple assays indicates the importance of systematically examining multiple functionalization strategies and architectures for potential applications such as direct viral neutralization.

This study points towards several steps that should be further addressed as the cTRP platform (and other self-assembling protein scaffolds) are further optimized. The mixed results from the assembly of the larger of the tcTRP constructs (harboring 24 repeats in various assemblages) appear to indicate that small design inaccuracies may be magnified as construct size increases and indicates that further refinement of the computational approach may yield additional improvement in behavior and performance. At the same time, the successful creation of self-assembling tcTRPs with increased surfaces and interface areas (and corresponding larger numbers of residues involved in the contacts within those interfaces) may facilitate yet another generation of self-assembling symmetric protein particles, in which homo-oligomeric assemblages are further diversified through the design and incorporation of unique contacts between each subunit (perhaps in the form of directional hydrogen-bond networks) that facilitate the creation of hetero-oligomeric particles and enable their functionalization with multiple forms of functional protein domains and cargo in precisely defined stoichiometric ratios and organizational patterns.

## Methods

### Constructs and nomenclature

The construct names, sequences, and figures for all constructs described in this paper are provided in Supplementary Table S1. The constructs described in this article (and listed in that Table) are referred to as ‘tcTRPs’ (‘thick circular Tandem Repeat Proteins’). Following the nomenclature for previous cTRP constructs described and used in a prior study^5^, the exact size and assemblages are further annotated as ‘tcTRP9’ and ‘tcTRP24’, respectively, where the underlying tcTRP9 and tcTRP24 scaffolds contain a total of 9 or 24 repeats. For scaffolds that are assembled from smaller identical protein subunits, the constructs are annotated as tcTRP9_*x*_ and tcTRP24_*x*_, where each subunit contains ‘*x*’ repeats. For example, ‘tcTRP9_3_’ (read as ‘tcTRP9 sub3’) refers to a particle containing a total of nine repeats, assembled from the trimerization of protein subunits containing 3 repeats each. Similarly, ‘tcTRP24_8_’ corresponds to a particle containing a total of 24 repeats, assembled from the trimerization of protein subunits containing eight repeats each_._ Some tcTRP24 constructs contain disulfide staples between protein subunits and are named ‘tcTRP24_*x*_SS*’*.

Finally, constructs harboring additional protein cargo fused at defined positions around the tcTRP periphery are further denoted in the form ‘tcTRP24SS-Cargo_*Y*_’, where ‘*y*’ is the total number of cargo domains per construct. One example in this paper (‘tcTRP24_8_-SH2_6_’) corresponds to a trimeric tcTRP containing a total of 24 repeats (‘tcTRP24…’), assembled from three identical subunits that contain 8 repeats each (‘tcTRP24_8_…’), and with each subunit further displaying two SH2 peptide-binding domains, for a total of six such domains displayed (‘tcTRP24_8_-SH2_6_’).

### Computational protein design

Protein design simulations were conducted exactly as described previously^5^. That approach corresponds to a geometry-guided repeat computational strategy implemented in the Rosetta package^18^ with additional de novo design elements^17^. Key features include the application of parametric symmetrization of backbone and side-chain conformations applied across all repeats (such that computational complexity scales only with repeat length); a pseudo-energy term that optimizes the inter-repeat geometry; clustering and resampling protocols that allow intensified exploration of promising topologies; and an *in silico* validation step that assesses sequence-structure compatibility by attempting to re-predict the designed structure given only the designed sequence. Applying this design procedure produces a diverse array of toroidal structures.

In this work, two additional modifications of the previously described approach were implemented: the ‘Ref2015’ energy function^31^ was used for all protein design and structure recapitulation calculations, and the range of allowed helix lengths was increased to 20−45 residues. Initial simulations explored helical linkers of length 1−5 residues with unconstrained backbone torsion angles. Clustering analysis of low-energy designs from these simulations revealed convergence on a 2-residue, antiparallel connection with backbone conformation ‘GB’ (one residue in a left-handed alpha-helical conformation and one residue in an extended conformation). A subsequent round of designs focused on ‘GB’ linkers was conducted to enhance sampling in this low-energy region of conformational space.

The identification of residue positions for the incorporation of disulfide staples into the tcTRP24_8_ trimer was performed by utilizing the Rosetta ‘Disulfidize Mover’ routine^32^. Each edge helix involved in the trimerization was selected and corresponding residues scanned. The distance between adjoining beta-carbons was used to determine potential residues; once identified they were mutated to cystine residues and tested through rotamer optimization and energy minimization.

### Protein expression and purification

All constructs encoding tcTRPs described in this study were designed and ligated into an in-house pET15HE expression vector^33^ or a commercially available pET28b expression vector and sequence verified. The coding sequence and the corresponding translated protein sequences, including the N-terminal poly-histidine affinity tag and thrombin cleavage site preceding the first tcTRP repeat, are provided in Supplementary Table S1. The free SH2 domain was subcloned and purified as previously described^5^.

Plasmids were transfected into BL21(DE3)-RIL *Escherichia coli* cells (Agilent Technologies) and plated on LB medium augmented with 100 μg mL^−1^ ampicillin. Protein was expressed via a previously described autoinduction protocol^34^. Briefly, 1 L of ZYP-5052 media containing 100 μg mL^−1^ ampicillin was inoculated with individual transformants, shaken at 37 °C for 8 h followed by 16 °C for 24 h. Expression cultures were pelleted by centrifugation and stored at −20 °C until purification.

Frozen cell pellets were thawed at room temperature and resuspended in 100 mL of 1× phosphate-buffered saline (‘PBS; 137 mM NaCl, 10 mM Na_2_HPO_4_, 2.7 mM KCl, pH 7.4.). PMSF was added to a final concentration of 0.5 μM. Cells were lysed via sonication and centrifuged in an SS34 rotor at 16,000 rpm for 20 min at 4 °C to remove cell debris. The supernatant was passed through a 5 μm filter, added to 2 mL of nickel-NTA metal affinity resin (Invitrogen) equilibrated with 1× PBS, and then incubated on a rocker platform at 4 °C for 1 h. After loading onto a gravity-fed column, the resin was washed twice with 25 mL of PBS containing 25 mM Imidazole. The protein was then eluted from the column by three additions of 5 mL 1× PBS containing 300 mM Imidazole. Fractions containing the eluted protein were pooled, concentrated, and buffer exchanged into 1× PBS. The sample was then filtered through a 0.2 μm filter and run over a size exclusion column (Cytiva HiLoad 16/60 Superdex 200) equilibrated in either 1× PBS or 20 mM Tris pH 7.5 + 150 mM NaCl.

Those constructs encoding tcTRPs trimers functionalized with three identical copies of VHH 72D61R (an anti-SARS-CoV-2 camelid nanobody targeting the viral receptor-binding domain or ‘RBD’)^23^ were subcloned into a modified pET28 b(+) vector where the Kanamycin bacterial resistance gene is replaced with Ampicillin bacterial resistance gene. The constructs were sequence verified by Sanger sequencing. Protein expression was carried out using the method described above for tcTRPs expression, with the additional step of buffer exchange via dialysis from Ni-NTA elution buffer to 1×PBS, prior to use in assays.

### Circular dichroism

Purified proteins were dialyzed overnight into 10 mM potassium phosphate buffer at pH 7.0, then diluted to 20.8 μM, as determined using the trimeric molecular weight of 166077 Daltons and extinction coefficient on a NanoDrop spectrophotometer (Thermo Fisher). Thermal denaturation experiments were performed on a JASCO J-815 spectrometer with a Peltier temperature controller. Wavelength scans from 190–250 nm were performed at 25 °C, 95 °C, and cooling back to 25 °C.

### Crystallization and structure determination of tcTRP9 and tcTRP9_3_

Both purified proteins were crystallized at 22 mg/mL with 100 mM sodium acetate pH 4.5 and 25% polyethylene glycol 400 in a 24-well hanging drop tray. Crystals were cryocooled in the same buffer via direct plunge into liquid nitrogen. Data was collected under cryocooled conditions (−150 °C) on a Saturn 944+ CCD area detector (Rigaku Inc.) using X-rays produced at 1.54 Å wavelength by a Rigaku HF-007 rotating anode generator. Data were processed using program HKL2000^35^. Molecular replacement was performed using the de novo designed model of tcTRP9 using PHASER^36^ in the PHENIX program suite^37^. Refinement was done utilizing programs COOT^38^ and REFMAC^39^. Figures were generated using program PYMOL^40^. The final Ramachandran distribution for backbone angles (favored, allowed, outliers) were 98.6, 1.4, 0% for tcTRP9 and 96.3, 2.5, 1.2% for tcTRP9_3_ (Table 1).

### Cryogenic electron microscopy (CryoEM) visualization of tcTRP24_8_ and tcTRP24_8_SS

Both purified proteins were screened with negative-stained transmission electron microscopy (TEM) using a 120 KV JOEL1400 electron microscope equipped with a 16 megapixel (4k × 4k) GATAN RIOL CMOS detector. The samples were prepared by depositing 4 μL of purified proteins at approximately 40 nM to the surface of a glow-discharged uniform carbon-coated grid. The particles were allowed to adsorb to the carbon film for ~ 1 min and washed three times with 20 ul of water and once with a drop of 0.7% uranyl formate followed by staining for 25 s with a 40 μL droplet of uranyl formate solution. Excess stains were wicked away with filter paper and the grids were air-dried overnight prior to analysis.

The tcTRP24_8_SS particles were further analyzed by CryoEM. Samples were prepared by applying an aliquot of 3 μL protein sample of tcTRP24_8_SS to a glow-discharged Quantifoil1.2/1.3 holey carbon grid, blotted with filter paper for 5 s and plunge-cooled in liquid ethane using an FEI Vitrobot Mark IV. Cryo-EM micrographs were collected on a 200 kV Glacios microscope (FEI) equipped with a Gatan K2 Summit direct detection camera. The microscope was operated at a calibrated magnification of 37,000×, yielding a pixel size of 1.16 Å on micrographs with an accumulated dosage of 60 e−/A^2^S. In total, 627 movies were collected from two screening sessions, including 82 at a tilt angle of 45°.

All data preprocessing, 2D classification, and 3D model generation and refinement, as well as post refinement polishing, were performed using the software package CryoSPARC2^41^. For each movie stack, the frames were aligned for beam-induced motion correction using Patch-motion-correction. Patch-CTF was used to estimate the contrast transfer function (CTF) parameter. A new ring-shape algorithm with inner/outer diameters of 100/120 was used for automated blob picking. After inspection and local motion correction, 627763 particles were accepted for reference-free 2D classification. Two consecutive runs of 2D classification/selection were used to root out false positive and bad (overlapping) particles. A total of 121426 particles in 20 classes were used for ab initio 3D reconstruction.

It is obvious from the selected classes that there were at least two populations of particles with different diameters. Three models were requested for ab initio 3D reconstruction. Results from 3D reconstruction showed multiple circular-disk particles with different diameters. The proportion of the three 3D classes varied with the number of consecutive 2D classification/select and images selected. Multiple trials were performed with different particle picking protocols and particle diameters. All approaches yielded similar results.

### Peptide binding assays in solution via fluorescence polarization

A 10-residue peptide, Tir10, containing a phosphorylated tyrosine (‘pY’), was chemically synthesized with a FITC tag at the 5′-end linked to the peptide with a 7 atom aminohexanoyl space, Ahx (GenScript).

$${{{{{\rm{Tir10}}}}}}:{{{{{\rm{FITC}}}}}}-{{{{{\rm{Ahx}}}}}}-{{{{{\rm{EHI}}}}}}({{{{{\rm{pY}}}}}}){{{{{\rm{DEVAAD}}}}}}$$

Tir10 stock was re-suspended to 5.7 mM in DMSO, then diluted to 0.5 μM in fluorescence polarization (‘FP’) Buffer (20 mM HEPES, 150 mM KCl, pH 7.4). Proteins were exchanged into FP Buffer then two-fold serially diluted from 23 to 0.01 μM (tcTRP24_8_SS and free SH2) or 34 to 0.02 μM (tcTRP24_8_SS-SH2_2_). Diluted proteins were mixed with Tir10 at a ratio of 9:1 for final concentrations of 21–0.01 μM or 31–0.015 μM protein, respectively, and 0.05 μM Tir10, then incubated, shielded from light, at room temperature for 20 min. FP values were read at excitation of 485 nm and emission of 525 nm (SpectraMax M5). After subtracting FP buffer only background from the raw perpendicular (*S*) and parallel (*P*) measurements, polarization (*mP*) and anisotropy (*r*) were calculated with the following equations:

$$mP=	 \, \left(\frac{P\,-\,S}{P\,+\,S}\right)\times 1000\\ r=	 \frac{P\,-\,S}{P\,+\,2S}$$

### Peptide binding assays on a surface via surface plasmon resonance (SPR)

SPR experiments were performed at 25 °C on a Biacore T100 instrument (Cytiva) with a Series S SA chip using a running buffer of 10 mM HEPES, pH 7.4, 150 mM NaCl, 3 mM EDTA, 0.05% surfactant P20 with 0.1 mg/mL bovine serum albumin. Biotinylated Tir-10_v2 (Biotin-Ahx-EHI-pY-DEVAAD) at 10 ng/mL was injected over one flow cell at a flow rate of 10 μL/minute to capture ~15 RUs of peptide. A flow cell with streptavidin alone was used as a reference surface. Analytes were repurified by SEC just prior to use. Buffer blanks and analytes (10 nM tcTRP24_8_SS, 60 nM free SH2, and 10 nM tcTRP24_8_SS-SH2_2_) were injected at a flow rate 50 μL/minute with 7 min of association and 10 min of dissociation. An overlay plot of double-referenced data was generated, then normalized for off-rate comparison by dividing each curve by its maximum response in Scrubber 2.0b software (BioLogic Software). Maximum binding responses observed were 125 and 123 RUs for free SH2 and tcTRP24_8_SS-SH2_2_, respectively. The tcTRP24_8_SS control did not bind. The figure was made in Prism 7 (GraphPad) for Mac OS X version 7.0d.

### SARS-CoV-2 pseudoviral neutralization assays

The SARS-CoV-2 pseudotyped lentiviral particle generation, tittering and neutralization assays were performed as previously described protocol^42^ with minor modifications, as described here. Poly-L-lysine coated clear bottom 96-well black plates (Thermo Scientific, 12-566-70) were seeded with 293-ACE2 cells (provided by the Jessie Bloom lab at the Fred Hutchinson Cancer Research Center) at a density of 1.25 × 10^4^ cells per well in 50 µL volume. Twelve hours after seeding, five-fold antibody dilutions, starting at 50 µg/mL, were prepared. Control, virus only and cell only samples were prepared as previously reported. 60 µL of the titered virus was added and mixed with antibody dilutions and virus only wells. The mix was incubated at 37 °C for 1 h. 100 µL of the mix from each well was added to the corresponding well on 293-ACE2 seeded plates. Polybrene (Sigma Aldrich, P4707) was added to each well at a final concentration of 5 µg/mL. Plates were incubated at 37 °C for 60 h post infection. Virus neutralization was assessed by measuring luminescence. While incubating, Bright-Glo Luciferase reagents (Promega, E2610) were thawed, equilibrated at room temperature, and prepared following the manufacturer’s recommendation. 100 µL of growth media was removed from each well and 30 µL per well of luciferase reagent added. Plates were incubated for 2 min at room temperature in the dark and luminescence was measured using an M2 plate reader (Molecular Devices). Luminescence RLUs from virus only wells were normalized as 100% infectivity and RLUs from cells only were normalized as 0% infectivity. Infectivity and IC50 were calculated using Four Parameter Logistic Regression on GraphPad Prism (GraphPad Software).

### ELISA based trimer VHH binding to SARS-CoV-2 RBD

To assay trimeric VHH binding to RBD, ELISA-based binding assays were performed using the following protocol. High-affinity ELISA plates (Greiner Bio-one, Catalog Number 655084) were coated with SARS-CoV-2 RBD (Roland Strong, Fred Hutchinson Cancer Research Center) at 1 µg/mL in bicarbonate buffer (Sigma, Catalog Number C3041-100CAP) overnight at 4 °C. Coated plates were washed three times with ELISA wash buffer (1XPBS, (Fisher BioReagents, Catalog Number B399-4), supplemented with 0.05% Tween-20 (Thermo Fisher Scientific, Catalog Number B2337-500)). ELISA plates were washed on AquaMax 2000 Microplate Washer (Molecular Devices). Washed plates were blocked with ELISA blocking buffer (ELISA wash buffer supplemented with 5% Non-fat dried milk) for 2 h at room temperature. While blocking, VHH dilutions were prepared as follows. Trimeric VHH 72D61R were diluted with ELISA blocking buffer in 4-fold serial dilution starting at 10 µg/mL. ELISA blocking solutions were aspirated, and 100 µL of antibody dilutions were added into each corresponding well. Plates were incubated at room temperature for 60 min and washed three times with ELISA wash buffer.

The detection antibody, HRP conjugated MonoRab^TM^ Rabbit Anti-Camelid VHH Cocktail (GenScript, A02016) was diluted 1:10,000 in ELISA blocking buffer. The detection antibody was added at 100 µL/well and plates were incubated for 30 min at room temperature then washed two times with ELISA wash buffer followed by a wash with 1×PBS. Peroxidase activity was measured using chemiluminescence by adding 100 µL Sera Care KPL TMB Microwell Peroxidase Substrate (Sera Care Life Sciences Inc., 5120-0047) following the manufacturer’s recommendation. Peroxidase activity was quenched after 5 min incubation at room temperature by adding 50 µL per well 1 M Hydrochloric acid. Chemiluminescence was measured on M2 plate reader (Molecular Devices) at 450 nm wavelength using SoftMax software (Molecular Devises). Binding EC50 was calculated using averages of replicates and Four Parameter Logistic Regression on GraphPad Prism (GraphPad Software).

### Bio-Layer Interferometry (BLI) based Trimer VHH binding to SARS-CoV-2 RBD

BLI measurements were performed on the Octet RED96 system (ForteBio) using High Precision Streptavidin (SAX) Biosensors (ForteBio). Biosensors were hydrated with phosphate-buffered saline (PBS) at pH 7.4 at room temperature for 10 min in 96-well flat-bottom microplate (Greiner, 655209). All kinetics experiments were performed at 30 °C with 1000 rpm agitation in the kinetics module. Biosensors were dipped into PBS containing wells for 60 s prior to antigen loading. Biosensors were loaded with enzymatically biotinylated RBD (provided by the Roland Strong Lab at the Fred Hutchinson Cancer Research Center) at 1 µg/mL in phosphate buffer, pH 7.4 for 300 s to achieve ~0.6–1 nm response. Loading was quenched by incubating biosensors in 50 µM Biocytin (Sigma Aldrich, 576-19-2) for 60 s. Baseline were established by incubating antigen-loaded biosensors in kinetics buffer (PBS + 0.02% Tween 20, 0.1% BSA, 0.05% Sodium azide) for 120 s. Following baseline measurements to determine the rate of association, antigen-loaded biosensor tips were dipped for 50 s into three-fold dilution series of trimeric tcTRP9_3_-VHH_3_ fusions starting at a protein concentration corresponding to approximately 100 nM. Analyte bound biosensors were dipped into kinetics buffer for 120–300 s to measure the rate of dissociation. Kinetic analyses were performed using the HT 11.1.1.39 Data Analysis module (ForteBio). Results were double referenced. The association and dissociation steps were both used in a 1:1 binding model with global fitting.

### Statistics and reproducibility

Biochemical experiments reporting binding interactions (flourescence polarization (FP) assays for the SH2-tcTRP fusions in Fig. 7; BLI and ELISA assays for VHH-tcTRP constructs in Fig. 8 and viral pseudoneutralization assays in Fig. 8) were conducted in triplicate using independent aliquots of each protein. Data are shown as mean and standard for *n* = 3 measurements.

### Reporting summary

Further information on research design is available in the Nature Research Reporting Summary linked to this article.

## Supplementary information

Supplemental Information

Reporting Summary

## Data Availability

The structures reported in this study have been deposited in the protein data base (www.rcsb.org) under PDB ID (entries 6XR1, 6XR2, and 7RDR) and the EMDB (entry 24425) and are available for immediate download. The original source data corresponding to all biochemical analyses have been added to the Harvard Dataverse depository (at https://dataverse.harvard.edu/dataverse/tcTRP) for public download and are also archived at our respective institutions and available upon request. Plasmids corresponding to tcTRP9 and tcTRP24 constructs described in this paper are available from the authors upon request and have also been provided to the Addgene public depository (IDs 175793 through 175800). Any remaining information can be obtained from the corresponding author upon reasonable request.

## Code availability

The use of all computational algorithms in this study is described in Methods and the corresponding code and programs are all available to the public.
