# Supplementary material for: Design of functionalised circular tandem repeat proteins with longer repeat topologies and enhanced subunit contact surfaces
Source: Commun Biol. 2021 Oct 29;4:1240. doi: 10.1038/s42003-021-02766-y (PMC8556268; doi:10.1038/s42003-021-02766-y)
Supplement: Supplementary file 1 — Supplemental Information [file 42003_2021_2766_MOESM1_ESM.pdf]

SUPPLEMENTARY INFORMATION

Supplemental Tables:

Table S1. Coding sequences, translated protein sequences and figures for constructs in this study

Table S2. Binding of trimeric tcTRP Anti-SARS CoV2 VHH domains to SARS RBD

Supplemental Figures:

Supplemental Figure S1 (**Related to Figures 1, 3 and 4).** Expression and purification of tcTRP9 and tcTRP9$_3$.

Supplemental Figure S2 (related to Figures 1, 3 and 4)**.** Comparison of the dimensions and topology of a 9 repeat 'cTRP' (circular Tandem Repeat Protein as described in[13]) compared to a comparable 9 repeat 'tcTRP' (thick circular Tandem Repeat Protein) described in this study

Supplemental Figure S3 (related to Figure 5). Expression of tcTRP24$_6$ and tcTRP24$_8$ and purification of tcTRP24$_8$.

Supplemental Figure S4 (Related to Figure 6). 2-D projections of tcTRP24$_8$SS used for CryoEM reconstruction (Figure 5) validates the overall design and dimensions of that construct.

Supplemental Figure S5 (Related to Figure 8 and Supplemental Table S2). Representative binding sensorgrams for determination of kinetics of binding interactions and equilibrium dissociation constants (K_D) for the interaction between homotrimeric tcTRP9$_3$-VHH$_3$ constructs and SARS-CoV2 RBD.

Table S1. Coding sequences, translated protein sequences and figures for constructs in this study

>tcTRP9 (Figures 1, 3, 4, S1, S2)

ATGGCTAGCAGCCATCATCATCATCATCATAGCAGCGGCCTGGTGCCGCGCGGCAGCTCCATGGGCAACCTGGAACTGGCAC
TGAAAGCTCTGCAGATTCTGGTGAATGCGGCCTATGTTCTGGCGGAAATCGCCCGTGATCGCGGTAACGAAGAACTGCTGGAA
AAAGCGGCTCGTCTGGCCGAAGAAGCGGCACGTCAGGCAGAAGAAATTGCACGTCAAGCTCGCAAAGAAGGCAACCTGGAAC
TGGCGCTGAAAGCCCTGCAAATTCTGGTCAATGCAGCTTACGTGCTGGCAGAAATCGCTCGTGACCGCGGTAATGAGGAGCTG
CTGGAAAAAGCGGCCCGTCTGGCAGAAGAAGCAGCTCGCCAGGCCGAAGAAATCGCGCGTCAAGCCCGTAAAGAAGGCAATC
TGGAACTGGCCCTGAAAGCCCTGCAAATCCTGGTTAATGCGGCCTATGTCCTGGCCGAAATTGCGCGCGACCGTGGCAATGAG
GAATTACTGGAAAAAGCGGCGCGCCTGGCCGAAGAAGCGGCCCGCCAAGCCGAAGAAATCGCCCGCCAGGCCCGTAAAGAA
GGCAACTTAGAGCTGGCTCTGAAAGCGCTGCAAATCCTGGTGAATGCAGCTTACGTTCTGGCCGAAATTGCCCGCGACCGCGG
TAATGAGGAACTTCTGGAAAAAGCCGCACGTCTGGCCGAAGAAGCAGCTCGTCAAGCGGAAGAAATTGCCCGTCAAGCCCGCA
AAGAAGGCAATTTAGAGCTGGCCCTGAAAGCGCTGCAGATTCTGGTCAATGCGGCCTATGTGCTGGCCGAAATTGCGCGTGAC
CGTGGCAATGAGGAACTCCTGGAAAAAGCGGCGCGTTTAGCCGAAGAAGCGGCCCGTCAAGCCGAAGAAATCGCTCGCCAGG
CCCGAAAAGAAGGCAATTTAGAATTAGCTTTAAAAGCGCTGCAAATTCTGGTGAACGCCGCTTACGTCCTGGCCGAAATTGCCC
GTGATCGCGGTAATGAGGAACTACTGGAAAAAGCCGCCCGCCTGGCCGAAGAAGCAGCCCGTCAGGCGGAAGAAATTGCTCG
CCAGGCCCGGAAAGAAGGCAATTTGGAGCTGGCCCTGAAAGCACTGCAAATCCTGGTCAACGCCGCGTACGTGCTGGCCGAA
ATCGCGCGCGACCGTGGCAACGAGGAGTTATTAGAAAAAGCGGCGCGTCTTGCCGAAGAAGCGGCGCGCCAGGCCGAAGAA
ATTGCGCGCCAGGCCAGAAAAGAAGGCAATCTTGAGCTGGCTCTGAAAGCTCTGCAAATCCTGGTCAATGCCGCCTACGTTCT
GGCAGAAATCGCGCGTGACCGCGGTAATGAAGAGTTACTGGAAAAAGCCGCGCGCTTAGCCGAAGAAGCAGCGCGCCAAGCC
GAAGAAATTGCGCGTCAAGCACGTAAAGAAGGCAACCTTGAGCTGGCACTGAAGGCGTTACAAATTCTGGTTAACGCGGCCTA
TGTGCTGGCGGAAATCGCGCGCGACCGCGGCAATGAGGAACTGTTAGAAAAAGCGGCACGCTTAGCCGAAGAAGCGGCGCG
TCAAGCCGAAGAAATTGCTCGCCAAGCACGCAAAGAAGGCTAA

**MASSHHHHHHSSGLVPRGSSMGNLELALKALQILVNAAYVLAEIARDRGNEELLEKAARLAEEAARQAEEIARQARKEGNLELAL
KALQILVNAAYVLAEIARDRGNEELLEKAARLAEEAARQAEEIARQARKEGNLELALKALQILVNAAYVLAEIARDRGNEELLEKAA
RLAEEAARQAEEIARQARKEGNLELALKALQILVNAAYVLAEIARDRGNEELLEKAARLAEEAARQAEEIARQARKEGNLELALKA
LQILVNAAYVLAEIARDRGNEELLEKAARLAEEAARQAEEIARQARKEGNLELALKALQILVNAAYVLAEIARDRGNEELLEKAARL
AEEAARQAEEIARQARKEGNLELALKALQILVNAAYVLAEIARDRGNEELLEKAARLAEEAARQAEEIARQARKEGNLELALKALQ
ILVNAAYVLAEIARDRGNEELLEKAARLAEEAARQAEEIARQARKEGNLELALKALQILVNAAYVLAEIARDRGNEELLEKAARLAE
EAARQAEEIARQARKEG**

>tcTRP9$_3$ (Figures 4, S1)

ATGGCTAGCAGCCATCATCATCATCATCATAGCAGCGGCCTGGTGCCGCGCGGCAGCTCCATGGGCAACCTGGAACTGGCAC
TGAAAGCTCTGCAGATTCTGGTGAATGCGGCCTATGTTCTGGCGGAAATCGCCCGTGATCGCGGTAACGAAGAACTGCTGGAA
AAAGCGGCTCGTCTGGCCGAAGAAGCGGCACGTCAGGCAGAAGAAATTGCACGTCAAGCTCGCAAAGAAGGCAACCTGGAAC
TGGCGCTGAAAGCCCTGCAAATTCTGGTCAATGCAGCTTACGTGCTGGCAGAAATCGCTCGTGACCGCGGTAATGAGGAGCTG
CTGGAAAAAGCCGCACGTCTGGCCGAAGAAGCAGCTCGTCAGGCCGAAGAAATCGCGCGTCAAGCCCGTAAAGAAGGCAATC
TGGAACTGGCCCTGAAAGCCCTGCAAATCCTGGTTAATGCGGCCTATGTCCTGGCAGAAATCGCGCGCGACCGTGGCAATGA
GGAATTACTGGAAAAAGCGGCCCGCCTGGCCGAAGAAGCGGCCCGTCAGGCCGAAGAAATTGCCCGTCAAGCACGCAAAGAA
GGCTAA

**MASSHHHHHHSSGLVPRGSSMGNLELALKALQILVNAAYVLAEIARDRGNEELLEKAARLAEEAARQAEEIARQARKEGNLELAL
KALQILVNAAYVLAEIARDRGNEELLEKAARLAEEAARQAEEIARQARKEGNLELALKALQILVNAAYVLAEIARDRGNEELLEKAA
RLAEEAARQAEEIARQARKEG**

>tcTRP24$_6$ (Figure 2, 3, S3)

ATGGCTAGCAGCCATCATCATCATCATCATAGCAGCGGCCTGGTGCCGCGCGGCAGCTCCATGGGTAACAGCGAGCTGGCGG
CGCGTATCCTGATCATTCTGTTCCAGCAACTGGTGGAGCTGGCGCGTCTGGCGATTGAAAGCGGCGACGAGGAACTGCTGCG
TCGTGTGAGCGAGTGGCTGGAGGAAGTTATCAAGGATATGCGTCGTGTGGTTGAACAGGCGCTGCGTGAGGGTAACAGCGAA
CTGGCGGCTCGTATCCTGATTATTCTGTTTCAGCAACTGGTTGAGCTGGCGCGTCTGGCGATAGAGAGCGGTGATGAAGAGTT
ATTACGTCGTGTGAGCGAATGGCTGGAGGAAGTTATTAAAGACATGCGTCGTGTTGTGGAGCAGGCGCTGCGCGAAGGTAACA
GCGAACTGGCGGCACGTATCCTGATAATTCTGTTTCAACAACTGGTGGAGCTGGCGCGTCTGGCGATCGAGAGCGGTGATGAA
GAATTATTACGTCGTGTGAGCGAGTGGCTGGAGGAAGTTATAAAAGACATGCGTCGTGTGGTGGAGCAGGCGCTGCGTGAAG
GTAACAGCGAACTGGCGGCGCGTATCCTGATAATTCTGTTCCAACAACTGGTTGAGCTGGCGCGTCTGGCGATAGAAAGCGGT
GATGAAGAGCTTTTACGTCGTGTGAGCGAGTGGCTGGAGGAAGTTATAAAGGACATGCGTCGTGTTGTTGAGCAGGCGCTGCG
CGAGGGTAACAGCGAACTGGCGGCCCGTATCCTGATAATTCTGTTCCAGCAACTGGTGGAGCTGGCGCGTCTGGCGATAGAAT
CTGGTGATGAAGAGTTACTTCGTCGTGTGAGCGAGTGGCTGGAGGAAGTTATAAAGGATATGCGTCGTGTTGTGGAACAGGCG
CTGCGCGAGGGTAACAGCGAACTGGCGGCACGTATCCTGATCATTCTGTTTCAGCAACTGGTTGAGCTGGCGCGTCTGGCGAT
AGAAAGTGGTGATGAAGAGCTTCTTCGTCGTGTGAGCGAGTGGCTGGAGGAAGTTATAAAGGATATGCGTCGTGTAGTTGAGC
AGGCGCTGCGTGAGGGCTAA

**MASSHHHHHHSSGLVPRGSSMGNSELAARILIILFQQLVELARLAIESGDEELLRRVSEWLEEVIKDMRRVVEQALREGNSELAARI
LIILFQQLVELARLAIESGDEELLRRVSEWLEEVIKDMRRVVEQALREGNSELAARILIILFQQLVELARLAIESGDEELLRRVSEWLE
EVIKDMRRVVEQALREGNSELAARILIILFQQLVELARLAIESGDEELLRRVSEWLEEVIKDMRRVVEQALREGNSELAARILIILFQQ
LVELARLAIESGDEELLRRVSEWLEEVIKDMRRVVEQALREGNSELAARILIILFQQLVELARLAIESGDEELLRRVSEWLEEVIKDM
RRVVEQALREG**

>tcTRP24$_8$ (Figures 2, 3, 5, 6, S3)

ATGGCTAGCAGCCATCATCATCATCATCATAGCAGCGGCCTGGTGCCGCGCGGCAGCTCCATGGGTAACAGCGAGCTGGCGG
CGCGTATCCTGATCATTCTGTTCCAGCAACTGGTGGAGCTGGCGCGTCTGGCGATTGAAAGCGGCGACGAGGAACTGCTGCG
TCGTGTGAGCGAGTGGCTGGAGGAAGTTATCAAGGATATGCGTCGTGTGGTTGAACAGGCGCTGCGTGAGGGTAACAGCGAA
CTGGCGGCTCGTATCCTGATTATTCTGTTTCAGCAACTGGTTGAGCTGGCGCGTCTGGCGATAGAGAGCGGTGATGAAGAGTT
ATTACGTCGTGTGAGCGAATGGCTGGAGGAAGTTATTAAAGACATGCGTCGTGTTGTGGAGCAGGCGCTGCGCGAAGGTAACA
GCGAACTGGCGGCACGTATCCTGATAATTCTGTTTCAACAACTGGTGGAGCTGGCGCGTCTGGCGATCGAGAGCGGTGATGAA
GAATTATTACGTCGTGTGAGCGAGTGGCTGGAGGAAGTTATAAAAGACATGCGTCGTGTGGTGGAGCAGGCGCTGCGTGAAG
GTAACAGCGAACTGGCGGCGCGTATCCTGATAATTCTGTTCCAACAACTGGTTGAGCTGGCGCGTCTGGCGATAGAAAGCGGT
GATGAAGAGCTTTTACGTCGTGTGAGCGAGTGGCTGGAGGAAGTTATAAAGGACATGCGTCGTGTTGTTGAGCAGGCGCTGCG
CGAGGGTAACAGCGAACTGGCGGCCCGTATCCTGATAATTCTGTTCCAGCAACTGGTGGAGCTGGCGCGTCTGGCGATAGAAT
CTGGTGATGAAGAGTTACTTCGTCGTGTGAGCGAGTGGCTGGAGGAAGTTATAAAGGATATGCGTCGTGTTGTGGAACAGGCG
CTGCGCGAGGGTAACAGCGAACTGGCGGCACGTATCCTGATCATTCTGTTTCAGCAACTGGTTGAGCTGGCGCGTCTGGCGAT
AGAAAGTGGTGATGAAGAGCTTCTTCGTCGTGTGAGCGAGTGGCTGGAGGAAGTTATAAAGGATATGCGTCGTGTAGTGGAGC
AGGCGCTGCGAGAGGGTAACAGCGAACTGGCGGCACGTATCCTGATTATTCTGTTCCAGCAACTGGTGGAGCTGGCGCGTCT
GGCGATAGAAAGTGGGGATGAAGAGTTACTCCGTCGTGTGAGCGAGTGGCTGGAGGAAGTTATAAAGGATATGCGTCGTGTC
GTGGAGCAGGCGCTGCGGGAGGGTAACAGCGAACTGGCGGCACGTATCCTGATAATTCTGTTCCAGCAACTGGTTGAGCTGG
CGCGTCTGGCGATAGAAAGTGGCGATGAAGAGTTACTACGTCGTGTGAGCGAGTGGCTGGAGGAAGTTATAAAGGATATGCGT
CGTGTAGTAGAACAGGCGCTGCGCGAGGGTTAA
**MASSHHHHHHSSGLVPRGSSMGNSELAARILIILFQQLVELARLAIESGDEELLRRVSEWLEEVIKDMRRVVEQALREGNSELAARI
LIILFQQLVELARLAIESGDEELLRRVSEWLEEVIKDMRRVVEQALREGNSELAARILIILFQQLVELARLAIESGDEELLRRVSEWLE
EVIKDMRRVVEQALREGNSELAARILIILFQQLVELARLAIESGDEELLRRVSEWLEEVIKDMRRVVEQALREGNSELAARILIILFQQ
LVELARLAIESGDEELLRRVSEWLEEVIKDMRRVVEQALREGNSELAARILIILFQQLVELARLAIESGDEELLRRVSEWLEEVIKDM
RRVVEQALREGNSELAARILIILFQQLVELARLAIESGDEELLRRVSEWLEEVIKDMRRVVEQALREGNSELAARILIILFQQLVELAR
LAIESGDEELLRRVSEWLEEVIKDMRRVVEQALREG***

>tcTRP24$_8$SS (Figures 5, 6, S4)

ATGGCTAGCAGCCATCATCATCATCATCATAGCAGCGGCCTGGTGCCGCGCGGCAGCTCCATGGGTAATAGCGAACTGGCGG
CGCGTTGCCTGATTATCCTGTTTCAGCAACTGGTGGAACTGGCGCGTCTGGCGATTGAGAGCGGCGATGAGGAACTGCTGCGT
CGTGTGAGCGAGTGGCTGGAGGAAGTTATTAAAGACATGCGTCGTGTGGTTGAGCAAGCGCTGCGTGAGGGTAACAGCGAGC
TGGCGGCGCGCATCCTGATCATTCTGTTCCAGCAACTGGTGGAGCTGGCGCGCCTGGCGATCGAAAGCGGCGACGAAGAGTT
ATTACGTCGTGTGAGCGAATGGCTGGAGGAAGTTATCAAAGATATGCGTCGTGTGGTTGAACAAGCGCTGCGCGAAGGTAACA
GCGAACTGGCGGCGCGCATTCTGATCATTCTGTTTCAGCAACTGGTTGAGCTGGCGCGTCTGGCGATCGAGAGCGGTGACGA
AGAGTTACTTCGTCGTGTGAGCGAGTGGTTAGAGGAAGTTATCAAGGATATGCGTCGTGTTGTGGAGCAAGCGCTGCGCGAAG
GCAATAGCGAGCTGGCGGCGCGTATTCTGATTATCCTGTTCCAACAACTGGTGGAGTTAGCTCGTCTGGCGATTGAAAGCGGC
GACGAAGAATTATTACGTCGTGTGAGCGAGTGGCTAGAGGAAGTTATTAAGGACATGCGTCGTGTTGTTGAACAAGCGCTGCG
TGAAGGCAACTCTGAGCTGGCGGCGCGTATCCTGATTATTCTGTTCCAACAACTGGTTGAGTTAGCTCGCCTGGCGATTGAGA
GCGGTGACGAAGAATTACTTCGTCGTGTGAGCGAGTGGCTTGAGGAAGTTATTAAAGATATGCGTCGTGTTGTTGAGCAGGCG
CTGCGCGAAGGCAACAGTGAATTAGCTGCGCGTATTCTGATTATTCTGTTTCAACAACTGGTGGAGCTTGCTCGCCTGGCGATC
GAGAGCGGCGACGAAGAGCTTTTACGTCGTGTGAGCGAGTGGCTCGAGGAAGTTATTAAGGATATGCGTCGTGTGGTGGAGC
AGGCGCTGCGCGAGGGCAATTCTGAGTTAGCTGCGCGCATTTTAATTATCTTATTCCAGCAACTGGTTGAGTTAGCGCGCCTGG
CGATTGAAAGCGGTGACGAAGAGCTTCTTCGTCGTGTGAGCGAGTGGCTAGAGGAAGTTATAAAGGATATGCGTCGTGTCGTT
GAGCAGGCGCTGCGTGAAGGCAATAGCGAACTGGCGTGCCGTATTCTGATCATCCTTTTTCAGCAACTGGTGGAGTTAGCGCG
TCTGGCGATCGAAAGCGGTGATGAAGAGCTGCTGCGTCGTGTTAGCGAGTGGCTGGAAGAAGTTATCAAGGACATGCGTCGT
GTGGTGGAACAGGCGCTGCGTGAGGGTTAA

**MASSHHHHHHSSGLVPRGSSMGNSELAARCLIILFQQLVELARLAIESGDEELLRRVSEWLEEVIKDMRRVVEQALREGNSELAAR
ILIILFQQLVELARLAIESGDEELLRRVSEWLEEVIKDMRRVVEQALREGNSELAARILIILFQQLVELARLAIESGDEELLRRVSEWLE
EVIKDMRRVVEQALREGNSELAARILIILFQQLVELARLAIESGDEELLRRVSEWLEEVIKDMRRVVEQALREGNSELAARILIILFQQ
LVELARLAIESGDEELLRRVSEWLEEVIKDMRRVVEQALREGNSELAARILIILFQQLVELARLAIESGDEELLRRVSEWLEEVIKDM
RRVVEQALREGNSELAARILIILFQQLVELARLAIESGDEELLRRVSEWLEEVIKDMRRVVEQALREGNSELACRILIILFQQLVELAR
LAIESGDEELLRRVSEWLEEVIKDMRRVVEQALREG***

>tcTRP24$_8$SS-Cap (Figure 5)

ATGGCTAGCAGCCATCATCATCATCATCATAGCAGCGGCCTGGTGCCGCGCGGCAGCTCCATGGGTGGTAACAGCGAGGAAG
CGGCGCGTAAGCTGATCATTCTGTTCCAGCAACTGGTGGAGAAAGCGCGTAAGGCGATTGAAAGCGGCGACGAGGAAGAGCT
GCGTCGTGTGAGCGAGGAACTGGAGGAAGTTATCAAGGATATGCGTCGTGTGGTTGAACAGGCGCTGCGTGAGGGTAACAGC
GAACTGGCGGCTCGTATCCTGATTATTCTGTTTCAGCAACTGGTTGAGCTGGCGCGTCTGGCGATAGAGAGCGGTGATGAAGA
GTTATTACGTCGTGTGAGCGAATGGCTGGAGGAAGTTATTAAAGACATGCGTCGTGTTGTGGAGCAGGCGCTGCGCGAAGGTA
ACAGCGAACTGGCGGCACGTATCCTGATAATTCTGTTTCAACAACTGGTGGAGCTGGCGCGTCTGGCGATCGAGAGCGGTGAT
GAAGAATTATTACGTCGTGTGAGCGAGTGGCTGGAGGAAGTTATAAAAGACATGCGTCGTGTGGTGGAGCAGGCGCTGCGTGA
AGGTAACAGCGAACTGGCGGCGCGTATCCTGATAATTCTGTTCCAACAACTGGTTGAGCTGGCGCGTCTGGCGATAGAAAGCG
GTGATGAAGAGCTTTTACGTCGTGTGAGCGAGTGGCTGGAGGAAGTTATAAAGGACATGCGTCGTGTTGTTGAGCAGGCGCTG
CGCGAGGGTAACAGCGAACTGGCGGCCCGTATCCTGATAATTCTGTTCCAGCAACTGGTGGAGCTGGCGCGTCTGGCGATAG
AATCTGGTGATGAAGAGTTACTTCGTCGTGTGAGCGAGTGGCTGGAGGAAGTTATAAAGGATATGCGTCGTGTTGTGGAACAG
GCGCTGCGCGAGGGTAACAGCGAACTGGCGGCACGTATCCTGATCATTCTGTTTCAGCAACTGGTTGAGCTGGCGCGTCTGG
CGATAGAAAGTGGTGATGAAGAGCTTCTTCGTCGTGTGAGCGAGTGGCTGGAGGAAGTTATAAAGGATATGCGTCGTGTAGTG
GAGCAGGCGCTGCGAGAGGGTAACAGCGAACTGGCGGCACGTATCCTGATTATTCTGTTCCAGCAACTGGTGGAGCTGGCGC
GTCTGGCGATAGAAAGTGGGGATGAAGAGTTACTCCGTCGTGTGAGCGAGTGGCTGGAGGAAGTTATAAAGGATATGCGTCGT
GTCGTGGAGCAGGCGCTGCGGGAGGGTAACAGCGAACTGGCGGCACGTATCCTGGAGATTCTGTTCCAGCAACTGGTTGAGC

TGGCGCGTCTGGCGAAGGAAAGTGGCGATGAAGAGTTACTACGTCGTGTGAGCGAGTGGCTGGAGGAAGTTAAGAAGGATAT
GCGTCGTGTAGAGGAACAGGCGAAACGCGAGGGTATTATATAA

MASSHHHHHHSSGLVPRGSSMGGNSEEAARKLIILFQQLVEKARKAIESGDEEELRRVSEELEEVIKDMRRVVEQALREGNSELAA
RILIILFQQLVELARLAIESGDEELLRRVSEWLEEVIKDMRRVVEQALREGNSELAARILIILFQQLVELARLAIESGDEELLRRVSEWL
EEVIKDMRRVVEQALREGNSELAARILIILFQQLVELARLAIESGDEELLRRVSEWLEEVIKDMRRVVEQALREGNSELAARILIILFQ
QLVELARLAIESGDEELLRRVSEWLEEVIKDMRRVVEQALREGNSELAARILIILFQQLVELARLAIESGDEELLRRVSEWLEEVIKD
MRRVVEQALREGNSELAARILIILFQQLVELARLAIESGDEELLRRVSEWLEEVIKDMRRVVEQALREGNSELAARILEILFQQLVEL
ARLAKESGDEELLRRVSEWLEEVKKDMRRVEEQAKREGII*

>Free Nck SH2 (Figure 7)

ATGTCCGAGTGGTACTACGGGAACGTGACGCGGCACCAGGCCGAGTGCGCCCTCAACGAGCGGGGCGTGGAGGGCGACTTC
CTCATTAGGGACAGCGAGTCCTCGCCCAGCGACTTCTCCGTGTCCCTTAAAGCGTCAGGGAAGAACAAACACTTCAAGGTGCA
GCTCGTGGACAATGTCTACTGCATTGGGCAGCGGCGCTTCCACACCATGGACGAGCTGGTGGAACACTACAAAAAGGCGCCC
ATCTTCACCAGCGAGCACGGGGAGAAGCTCTACCTCGTCAGGGCCCTGCAGTGA

MSEWYYGNVTRHQAECALNERGVEGDFLIRDSESSPSDFSVSLKASGKNKHFKVQLVDNVYCIGQRRFHTMDELVEHYKKAPIFT
SEHGEKLYLVRALQ*

SH2 and VHH fusions to tcTRPs (inserted sequences are underlined; linker is *grey italicized*).

>tcTRP24$_8$-SH2$_6$ (Figure 7)

ATGGCTAGCAGCCATCATCATCATCATCATAGCAGCGGCCTGGTGCCGCGCGGCAGCTCCATGGGCAACAGCGAGCTGGCGG
CGCGTATCCTGATCATTCTGTTCCAGCAACTGGTGGAGCTGGCGCGTCTGGCGATTGAAAGCGGTGACGAGGAACTGCTGCGT
CGTGTGAGCGAGTGGCTGGAGGAAGTTATCAAGGATATGCGTCGTGTGGTTGAACAGGCGCTGCGTGAGGGTAACAGCGAAC
TGGCTGCTCGTATCCTGATTATCCTGTTTCAGCAACTGGTTGAGCTGGCGCGCCTGGCGATTGAAAGCGGCGATGAAGAACTG
TTACGTCGTGTGAGCGAATGGCTGGAGGAAGTTATTAAAGACATGCGTCGTGTTGTTGAGCAGGCGCTGCGTGAAGGTGGCG
GTAGCGACGGTCAGGATAAGGGTAGCGACGGCCAAGATAAAGGCGGTAGCGAGTGGTACTATGGCAACGTGACCCGTCACCA
AGCGGAATGCGCGCTGAACGAGCGTGGTGTTGAAGGCGACTTCCTGATCCGTGATAGCGAAAGCAGCCCGAGCGACTTTAGC
GTGAGCCTGAAGGCGAGCGGCAAGAACAAACACTTCAAAGTGCAGCTGGTTGACAACGTGTACTGCATCGGCCAACGTCGTTT
TCACACGATGGACGAGCTGGTTGAGCACTACAAGAAAGCGCCGATTTTCACCAGCGAGCACGGTGAAAAGCTGTATCTGGTTC
GTGCGCTGCAGGGCGGTAGCGACGGTCAAGACAAAGGTAGCGACGGCCAGGATAAAGGCAACAGCGAGCTGGCTGCTCGTA
TCCTGATAATCCTGTTTCAACAACTGGTGGAGCTGGCTCGCCTGGCGATCGAGAGCGGCGATGAGGAACTGTTACGTCGTGTG
AGCGAGTGGCTGGAGGAAGTTATAAAAGACATGCGTCGTGTGGTGGAGCAAGCGCTGCGCGAAGGTAACAGCGAACTGGCCG
CTCGTATCCTGATTATTCTGTTTCAACAACTGGTTGAGCTGGCTCGTCTGGCGATCGAGAGCGGTGATGAAGAACTGCTTCGTC
GTGTGAGCGAGTGGCTGGAGGAAGTTATAAAGGACATGCGTCGTGTTGTGGAGCAGGCGCTGCGCGAGGGTAACAGCGAACT
GGCAGCGCGTATCCTGATTATCTTATTTCAGCAACTGGTGGAGCTGGCACGCCTGGCGATCGAATCTGGTGATGAAGAACTGC
TCCGTCGTGTGAGCGAGTGGCTGGAGGAAGTTATAAAGGATATGCGTCGTGTTGTGGAACAGGCGCTGCGCGAGGGTAACAG
CGAACTGGCGGCTCGTATCCTGATCATCCTGTTCCAACAACTGGTTGAGCTGGCACGCCTGGCGATTGAGAGCGGCGATGAG
GAACTGCTTCGTCGTGTGAGCGAGTGGCTGGAGGAAGTTATAAAGGATATGCGTCGTGTAGTGGAACAGGCGCTGCGTGAAG
GCGGTGGCAGCGATGGCCAAGACAAGGGTAGCGATGGTCAAGATAAAGGTGGCAGCGAATGGTATTACGGTAATGTTACCCG
CCATCAGGCGGAGTGCGCGCTGAATGAACGTGGTGTTGAGGGTGACTTCCTGATTCGCGACTCTGAAAGCAGCCCTAGCGAC
TTTAGCGTTAGCCTGAAGGCGAGCGGCAAAAACAAACACTTTAAAGTTCAGCTGGTTGACAACGTTTATTGCATTGGTCAACGT
CGTTTCCATACGATGGACGAGCTGGTTGAGCATTATAAGAAAGCGCCGATCTTTACCAGCGAACATGGTGAAAAACTGTACCTG
GTGCGTGCGCTGCAGGGTGGCAGCGACGGTCAAGACAAGGGTAGCGACGGTCAAGATAAGGGCAACAGCGAGCTGGCCGCT
CGTATCCTGATAATTCTGTTTCAGCAACTGGTGGAGCTGGCCCGATTAGCGATCGAGAGCGGCGACGAAGAACTGCTACGTCG
TGTGAGCGAGTGGCTGGAGGAAGTTATAAAGGATATGCGTCGTGTCGTTGAACAAGCGCTGCGAGAAGGTAACAGCGAACTG
GCAGCACGTATCCTGATAATATTATTCCAGCAACTGGTTGAGCTGGCCCGTTTAGCTATTGAAAGCGGCGACGAGGAACTGTTA
CGTCGTGTGAGCGAGTGGCTGGAGGAAGTTATAAAGGATATGCGTCGTGTAGTAGAACAGGCGCTGCGCGAGGGTTAA

MASSHHHHHHSSGLVPRGSSMGNSELAARILIILFQQLVELARLAIESGDEELLRRVSEWLEEVIKDMRRVVEQALREGNSELAARI
LIILFQQLVELARLAIESGDEELLRRVSEWLEEVIKDMRRVVEQALRE*GGGS*DGQDKGSDGQDKGGSEWYYGNVTRHQAECALNE
RGVEGDFLIRDSESSPSDFSVSLKASGKNKHFKVQLVDNVYCIGQRRFHTMDELVEHYKKAPIFTSEHGEKLYLVRALQGGSDGQ
DKGSDGQDKGNSELAARILIILFQQLVELARLAIESGDEELLRRVSEWLEEVIKDMRRVVEQALREGNSELAARILIILFQQLVELARL
AIESGDEELLRRVSEWLEEVIKDMRRVVEQALREGNSELAARILIILFQQLVELARLAIESGDEELLRRVSEWLEEVIKDMRRVVEQA
LREGNSELAARILIILFQQLVELARLAIESGDEELLRRVSEWLEEVIKDMRRVVEQALRE*GGGS*DGQDKGSDGQDKGGSEWYYGN
VTRHQAECALNERGVEGDFLIRDSESSPSDFSVSLKASGKNKHFKVQLVDNVYCIGQRRFHTMDELVEHYKKAPIFTSEHGEKLYL
VRALQGGSDGQDKGSDGQDKGNSELAARILIILFQQLVELARLAIESGDEELLRRVSEWLEEVIKDMRRVVEQALREGNSELAARIL
IILFQQLVELARLAIESGDEELLRRVSEWLEEVIKDMRRVVEQALREG*

>tcTRP9$_3$-VHH678$_3$ (Figure 8)

ATGGGTAATCTTGAACTTGCATTGAAAGCACTGCAAATTCTTGTGAATGCTGCTTATGTATTAGCTGAGATTGCACGCGATCGTG
GCAACGAAGAATTGTTGGAGAAGGCGGCGCGTCTTGCTGAAGAAGCTGCCCGTCAAGCGGAAGAAATCGCGCGTCAGGCTCG
TAAGGAAGGCAATTTAGAGTTGGCGCTGAAAGCTTTGCAGATTTTAGTAAACGCGGCGTACGTCCTTGCTGAGATCGCCCGTGA
CCGCGGCAATGAGGAGCTTTTAGAGAAAGCGGCACGTCTTGCGGAGGAAGCTGCGCGTCAGGCAGAAGAAATTGCGCGCCAA
GCTCGTAAAGAAGGCAACTTGGAGCTTGCATTAAAGGCCCTTCAAATCCTGGTCAACGCTGCATATGTTCTGGCAGAAATTGCG
CGCGATCGCGGAAATGAAGAACTGCTTGAGAAAGCAGCGCGCCTTGCTGAGGAAGCAGCCCGCCAGGCGGAGGAGATTGCC
CGTCAGGCGCGCAAGGAAGGAAGCCCGGCCCCCGCCCCCGCTGGACAAGTACAACTTCAAGAAAGCGGCGGTGGGTTGGTT

CAGGCGGGCGGCAGCCTTCGTTTGTCCTGTGCCGCCTCTGGCCGTACCTTTAGTGAGTACGCAATGGGTTGGTTTCGCCAGG
CCCCTGGTAAGGAACGTGAATTTGTTGCAACTATCTCATGGAGTGGTGGCTCTACTTATTACACCCGTTCAGTAAAAGGACGTTT
CACTATCTCCCGCGACAACGCGAAAAACACCGTCTACTTGCAAATGAATAGCCTGAAGCCCGACGATACAGCAGTATATTATTG
CGCGGCAGCGGGGTTAGGAACAGTAGTATCAGAGTGGGATTACGACTACGACTATTGGGGACAGGGCACTCAAGTAACAGTG
TCAAGCGGACATCATCATCACCACCAC

**MGQVQLQESGGGLVQAGGSLRLSCAASGRTFSEYAMGWFRQAPGKEREFVATISWSGGSTYYTRSVKGRFTISRDNAKNTVYLQ
MNSLKPDDTAVYYCAAAGLGTVVSEWDYDYDYWGQGTQVTVSS*EAAAKGGG*NLELALKALQILVNAAYVLAEIARDRGNEELLE
KAARLAEEAARQAEEIARQARKEGNLELALKALQILVNAAYVLAEIARDRGNEELLEKAARLAEEAARQAEEIARQARKEGNLELA
LKALQILVNAAYVLAEIARDRGNEELLEKAARLAEEAARQAEEIARQARKEGGHHHHHH**

>tcTRP9$_3$-VHH679$_3$ (Figure 8)

ATGGGACAAGTACAACTTCAAGAAAGCGGCGGTGGGTTGGTTCAGGCGGGCGGCAGCCTTCGTTTGTCCTGTGCCGCCTCTG
GCCGTACCTTTAGTGAGTACGCAATGGGTTGGTTTCGCCAGGCCCCTGGTAAGGAACGTGAATTTGTTGCAACTATCTCATGGA
GTGGTGGCTCTACTTATTACACCCGTTCAGTAAAAGGACGTTTCACTATCTCCCGCGACAACGCGAAAAACACCGTCTACTTGC
AAATGAATAGCCTGAAGCCCGACGATACAGCAGTATATTATTGCGCGGCAGCGGGGTTAGGAACAGTAGTATCAGAGTGGGAT
TACGACTACGACTATTGGGGACAGGGCACTCAAGTAACAGTGTCAAGCCCGGCCCCCGCCCCCGCTGGAGGTAATCTTGAACT
TGCATTGAAAGCACTGCAAATTCTTGTGAATGCTGCTTATGTATTAGCTGAGATTGCACGCGATCGTGGCAACGAAGAATTGTTG
GAGAAGGCGGCGCGTCTTGCTGAAGAAGCTGCCCGTCAAGCGGAAGAAATCGCGCGTCAGGCTCGTAAGGAAGGCAATTTAG
AGTTGGCGCTGAAAGCTTTGCAGATTTTAGTAAACGCGGCGTACGTCCTTGCTGAGATCGCCCGTGACCGCGGCAATGAGGAG
CTTTTAGAGAAAGCGGCACGTCTTGCGGAGGAAGCTGCGCGTCAGGCAGAAGAAATTGCGCGCCAAGCTCGTAAAGAAGGCA
ACTTGGAGCTTGCATTAAAGGCCCTTCAAATCCTGGTCAACGCTGCATATGTTCTGGCAGAAATTGCGCGCGATCGCGGAAATG
AAGAACTGCTTGAGAAAGCAGCGCGCCTTGCTGAGGAAGCAGCCCGCCAGGCGGAGGAGATTGCCCGTCAGGCGCGCAAGG
AAGGAGGACATCATCATCACCACCAC

**MGQVQLQESGGGLVQAGGSLRLSCAASGRTFSEYAMGWFRQAPGKEREFVATISWSGGSTYYTRSVKGRFTISRDNAKNTVYLQ
MNSLKPDDTAVYYCAAAGLGTVVSEWDYDYDYWGQGTQVTVSS*PAPAPAGG*NLELALKALQILVNAAYVLAEIARDRGNEELLE
KAARLAEEAARQAEEIARQARKEGNLELALKALQILVNAAYVLAEIARDRGNEELLEKAARLAEEAARQAEEIARQARKEGNLELA
LKALQILVNAAYVLAEIARDRGNEELLEKAARLAEEAARQAEEIARQARKEGGHHHHHH**

>tcTRP9$_3$-VHH680$_3$ (Figure 8)

ATGGGACAAGTACAACTTCAAGAAAGCGGCGGTGGGTTGGTTCAGGCGGGCGGCAGCCTTCGTTTGTCCTGTGCCGCCTCTG
GCCGTACCTTTAGTGAGTACGCAATGGGTTGGTTTCGCCAGGCCCCTGGTAAGGAACGTGAATTTGTTGCAACTATCTCATGGA
GTGGTGGCTCTACTTATTACACCCGTTCAGTAAAAGGACGTTTCACTATCTCCCGCGACAACGCGAAAAACACCGTCTACTTGC
AAATGAATAGCCTGAAGCCCGACGATACAGCAGTATATTATTGCGCGGCAGCGGGGTTAGGAACAGTAGTATCAGAGTGGGAT
TACGACTACGACTATTGGGGACAGGGCACTCAAGTAACAGTGTCAAGCGCACCTGAGTTCCTTGGGGGACCTGGAGGTAATCT
TGAACTTGCATTGAAAGCACTGCAAATTCTTGTGAATGCTGCTTATGTATTAGCTGAGATTGCACGCGATCGTGGCAACGAAGA
ATTGTTGGAGAAGGCGGCGCGTCTTGCTGAAGAAGCTGCCCGTCAAGCGGAAGAAATCGCGCGTCAGGCTCGTAAGGAAGGC
AATTTAGAGTTGGCGCTGAAAGCTTTGCAGATTTTAGTAAACGCGGCGTACGTCCTTGCTGAGATCGCCCGTGACCGCGGCAA
TGAGGAGCTTTTAGAGAAAGCGGCACGTCTTGCGGAGGAAGCTGCGCGTCAGGCAGAAGAAATTGCGCGCCAAGCTCGTAAA
GAAGGCAACTTGGAGCTTGCATTAAAGGCCCTTCAAATCCTGGTCAACGCTGCATATGTTCTGGCAGAAATTGCGCGCGATCG
CGGAAATGAAGAACTGCTTGAGAAAGCAGCGCGCCTTGCTGAGGAAGCAGCCCGCCAGGCGGAGGAGATTGCCCGTCAGGC
GCGCAAGGAAGGAGGACATCATCATCACCACCAC

**MGQVQLQESGGGLVQAGGSLRLSCAASGRTFSEYAMGWFRQAPGKEREFVATISWSGGSTYYTRSVKGRFTISRDNAKNTVYLQ
MNSLKPDDTAVYYCAAAGLGTVVSEWDYDYDYWGQGTQVTVSS*APEFLGGPGG*NLELALKALQILVNAAYVLAEIARDRGNEEL
LEKAARLAEEAARQAEEIARQARKEGNLELALKALQILVNAAYVLAEIARDRGNEELLEKAARLAEEAARQAEEIARQARKEGNLE
LALKALQILVNAAYVLAEIARDRGNEELLEKAARLAEEAARQAEEIARQARKEGGHHHHHH**

>tcTRP9$_3$-VHH681$_3$ (Figure 8)

ATGGGACAAGTACAACTTCAAGAAAGCGGCGGTGGGTTGGTTCAGGCGGGCGGCAGCCTTCGTTTGTCCTGTGCCGCCTCTG
GCCGTACCTTTAGTGAGTACGCAATGGGTTGGTTTCGCCAGGCCCCTGGTAAGGAACGTGAATTTGTTGCAACTATCTCATGGA
GTGGTGGCTCTACTTATTACACCCGTTCAGTAAAAGGACGTTTCACTATCTCCCGCGACAACGCGAAAAACACCGTCTACTTGC
AAATGAATAGCCTGAAGCCCGACGATACAGCAGTATATTATTGCGCGGCAGCGGGGTTAGGAACAGTAGTATCAGAGTGGGAT
TACGACTACGACTATTGGGGACAGGGCACTCAAGTAACAGTGTCAAGCGCCCCGCCTCCCGTTCCTTCAACCCCCCCCACCCC
AAGTCCGTCAGCTGGAGGTAATCTTGAACTTGCATTGAAAGCACTGCAAATTCTTGTGAATGCTGCTTATGTATTAGCTGAGATT
GCACGCGATCGTGGCAACGAAGAATTGTTGGAGAAGGCGGCGCGTCTTGCTGAAGAAGCTGCCCGTCAAGCGGAAGAAATCG
CGCGTCAGGCTCGTAAGGAAGGCAATTTAGAGTTGGCGCTGAAAGCTTTGCAGATTTTAGTAAACGCGGCGTACGTCCTTGCT
GAGATCGCCCGTGACCGCGGCAATGAGGAGCTTTTAGAGAAAGCGGCACGTCTTGCGGAGGAAGCTGCGCGTCAGGCAGAA
GAAATTGCGCGCCAAGCTCGTAAAGAAGGCAACTTGGAGCTTGCATTAAAGGCCCTTCAAATCCTGGTCAACGCTGCATATGTT
CTGGCAGAAATTGCGCGCGATCGCGGAAATGAAGAACTGCTTGAGAAAGCAGCGCGCCTTGCTGAGGAAGCAGCCCGCCAGG
CGGAGGAGATTGCCCGTCAGGCGCGCAAGGAAGGAGGACATCATCATCACCACCAC

**MGQVQLQESGGGLVQAGGSLRLSCAASGRTFSEYAMGWFRQAPGKEREFVATISWSGGSTYYTRSVKGRFTISRDNAKNTVYLQ
MNSLKPDDTAVYYCAAAGLGTVVSEWDYDYDYWGQGTQVTVSS*APPPVPSTPPTPSPSAGG*NLELALKALQILVNAAYVLAEIAR
DRGNEELLEKAARLAEEAARQAEEIARQARKEGNLELALKALQILVNAAYVLAEIARDRGNEELLEKAARLAEEAARQAEEIARQA
RKEGNLELALKALQILVNAAYVLAEIARDRGNEELLEKAARLAEEAARQAEEIARQARKEGGHHHHHH**

>tcTRP9$_3$-VHH682$_3$ (Figure 8)

ATGGGTAATCTTGAACTTGCATTGAAAGCACTGCAAATTCTTGTGAATGCTGCTTATGTATTAGCTGAGATTGCACGCGATCGTG
GCAACGAAGAATTGTTGGAGAAGGCGGCGCGTCTTGCTGAAGAAGCTGCCCGTCAAGCGGAAGAAATCGCGCGTCAGGCTCG
TAAGGAAGGCAATTTAGAGTTGGCGCTGAAAGCTTTGCAGATTTTAGTAAACGCGGCGTACGTCCTTGCTGAGATCGCCCGTGA
CCGCGGCAATGAGGAGCTTTTAGAGAAAGCGGCACGTCTTGCGGAGGAAGCTGCGCGTCAGGCAGAAGAAATTGCGCGCCAA
GCTCGTAAAGAAGGCAACTTGGAGCTTGCATTAAAGGCCCTTCAAATCCTGGTCAACGCTGCATATGTTCTGGCAGAAATTGCG
CGCGATCGCGGAAATGAAGAACTGCTTGAGAAAGCAGCGCGCCTTGCTGAGGAAGCAGCCCGCCAGGCGGAGGAGATTGCC
CGTCAGGCGCGCAAGGAAGGTGGACAAGTACAACTTCAAGAAAGCGGCGGTGGGTTGGTTCAGGCGGGCGGCAGCCTTCGTT
TGTCCTGTGCCGCCTCTGGCCGTACCTTTAGTGAGTACGCAATGGGTTGGTTTCGCCAGGCCCCTGGTAAGGAACGTGAATTT
GTTGCAACTATCTCATGGAGTGGTGGCTCTACTTATTACACCCGTTCAGTAAAAGGACGTTTCACTATCTCCCGCGACAACGCG
AAAAACACCGTCTACTTGCAAATGAATAGCCTGAAGCCCGACGATACAGCAGTATATTATTGCGCGGCAGCGGGGTTAGGAAC
AGTAGTATCAGAGTGGGATTACGACTACGACTATTGGGGACAGGGCACTCAAGTAACAGTGTCAAGCGGACATCATCATCACC
ACCAC

**MGNLELALKALQILVNAAYVLAEIARDRGNEELLEKAARLAEEAARQAEEIARQARKEGNLELALKALQILVNAAYVLAEIARDRGN
EELLEKAARLAEEAARQAEEIARQARKEGNLELALKALQILVNAAYVLAEIARDRGNEELLEKAARLAEEAARQAEEIARQARKEG
GQVQLQESGGGLVQAGGSLRLSCAASGRTFSEYAMGWFRQAPGKEREFVATISWSGGSTYYTRSVKGRFTISRDNAKNTVYLQM
NSLKPDDTAVYYCAAAGLGTVVSEWDYDYDYWGQGTQVTVSSGHHHHHH**

>tcTRP9$_3$-VHH683$_3$ (Figure 8)

ATGGGTAATCTTGAACTTGCATTGAAAGCACTGCAAATTCTTGTGAATGCTGCTTATGTATTAGCTGAGATTGCACGCGATCGTG
GCAACGAAGAATTGTTGGAGAAGGCGGCGCGTCTTGCTGAAGAAGCTGCCCGTCAAGCGGAAGAAATCGCGCGTCAGGCTCG
TAAGGAAGGCAATTTAGAGTTGGCGCTGAAAGCTTTGCAGATTTTAGTAAACGCGGCGTACGTCCTTGCTGAGATCGCCCGTGA
CCGCGGCAATGAGGAGCTTTTAGAGAAAGCGGCACGTCTTGCGGAGGAAGCTGCGCGTCAGGCAGAAGAAATTGCGCGCCAA
GCTCGTAAAGAAGGCAACTTGGAGCTTGCATTAAAGGCCCTTCAAATCCTGGTCAACGCTGCATATGTTCTGGCAGAAATTGCG
CGCGATCGCGGAAATGAAGAACTGCTTGAGAAAGCAGCGCGCCTTGCTGAGGAAGCAGCCCGCCAGGCGGAGGAGATTGCC
CGTCAGGCGCGCAAGGAAGGAAGCGAGGCAGCCGCAAAGGGTGGACAAGTACAACTTCAAGAAAGCGGCGGTGGGTTGGTT
CAGGCGGGCGGCAGCCTTCGTTTGTCCTGTGCCGCCTCTGGCCGTACCTTTAGTGAGTACGCAATGGGTTGGTTTCGCCAGG
CCCCTGGTAAGGAACGTGAATTTGTTGCAACTATCTCATGGAGTGGTGGCTCTACTTATTACACCCGTTCAGTAAAAGGACGTTT
CACTATCTCCCGCGACAACGCGAAAAACACCGTCTACTTGCAAATGAATAGCCTGAAGCCCGACGATACAGCAGTATATTATTG
CGCGGCAGCGGGGTTAGGAACAGTAGTATCAGAGTGGGATTACGACTACGACTATTGGGGACAGGGCACTCAAGTAACAGTG
TCAAGCGGACATCATCATCACCACCAC

**MGNLELALKALQILVNAAYVLAEIARDRGNEELLEKAARLAEEAARQAEEIARQARKEGNLELALKALQILVNAAYVLAEIARDRGN
EELLEKAARLAEEAARQAEEIARQARKEGNLELALKALQILVNAAYVLAEIARDRGNEELLEKAARLAEEAARQAEEIARQARKEG
*SEAAAKG*GQVQLQESGGGLVQAGGSLRLSCAASGRTFSEYAMGWFRQAPGKEREFVATISWSGGSTYYTRSVKGRFTISRDNA
KNTVYLQMNSLKPDDTAVYYCAAAGLGTVVSEWDYDYDYWGQGTQVTVSSGHHHHHH**

>tcTRP9$_3$-VHH684$_3$ (Figure 8)

ATGGGTAATCTTGAACTTGCATTGAAAGCACTGCAAATTCTTGTGAATGCTGCTTATGTATTAGCTGAGATTGCACGCGATCGTG
GCAACGAAGAATTGTTGGAGAAGGCGGCGCGTCTTGCTGAAGAAGCTGCCCGTCAAGCGGAAGAAATCGCGCGTCAGGCTCG
TAAGGAAGGCAATTTAGAGTTGGCGCTGAAAGCTTTGCAGATTTTAGTAAACGCGGCGTACGTCCTTGCTGAGATCGCCCGTGA
CCGCGGCAATGAGGAGCTTTTAGAGAAAGCGGCACGTCTTGCGGAGGAAGCTGCGCGTCAGGCAGAAGAAATTGCGCGCCAA
GCTCGTAAAGAAGGCAACTTGGAGCTTGCATTAAAGGCCCTTCAAATCCTGGTCAACGCTGCATATGTTCTGGCAGAAATTGCG
CGCGATCGCGGAAATGAAGAACTGCTTGAGAAAGCAGCGCGCCTTGCTGAGGAAGCAGCCCGCCAGGCGGAGGAGATTGCC
CGTCAGGCGCGCAAGGAAGGAAGCCCGGCCCCCGCCCCCGCTGGACAAGTACAACTTCAAGAAAGCGGCGGTGGGTTGGTT
CAGGCGGGCGGCAGCCTTCGTTTGTCCTGTGCCGCCTCTGGCCGTACCTTTAGTGAGTACGCAATGGGTTGGTTTCGCCAGG
CCCCTGGTAAGGAACGTGAATTTGTTGCAACTATCTCATGGAGTGGTGGCTCTACTTATTACACCCGTTCAGTAAAAGGACGTTT
CACTATCTCCCGCGACAACGCGAAAAACACCGTCTACTTGCAAATGAATAGCCTGAAGCCCGACGATACAGCAGTATATTATTG
CGCGGCAGCGGGGTTAGGAACAGTAGTATCAGAGTGGGATTACGACTACGACTATTGGGGACAGGGCACTCAAGTAACAGTG
TCAAGCGGACATCATCATCACCACCAC

**MGNLELALKALQILVNAAYVLAEIARDRGNEELLEKAARLAEEAARQAEEIARQARKEGNLELALKALQILVNAAYVLAEIARDRGN
EELLEKAARLAEEAARQAEEIARQARKEGNLELALKALQILVNAAYVLAEIARDRGNEELLEKAARLAEEAARQAEEIARQARKEG
*SPAPAPA*GQVQLQESGGGLVQAGGSLRLSCAASGRTFSEYAMGWFRQAPGKEREFVATISWSGGSTYYTRSVKGRFTISRDNAK
NTVYLQMNSLKPDDTAVYYCAAAGLGTVVSEWDYDYDYWGQGTQVTVSSGHHHHHH**

>tcTRP9$_3$-VHH686$_3$ (Figure 8)

ATGGGTAATCTTGAACTTGCATTGAAAGCACTGCAAATTCTTGTGAATGCTGCTTATGTATTAGCTGAGATTGCACGCGATCGTG
GCAACGAAGAATTGTTGGAGAAGGCGGCGCGTCTTGCTGAAGAAGCTGCCCGTCAAGCGGAAGAAATCGCGCGTCAGGCTCG
TAAGGAAGGCAATTTAGAGTTGGCGCTGAAAGCTTTGCAGATTTTAGTAAACGCGGCGTACGTCCTTGCTGAGATCGCCCGTGA
CCGCGGCAATGAGGAGCTTTTAGAGAAAGCGGCACGTCTTGCGGAGGAAGCTGCGCGTCAGGCAGAAGAAATTGCGCGCCAA
GCTCGTAAAGAAGGCAACTTGGAGCTTGCATTAAAGGCCCTTCAAATCCTGGTCAACGCTGCATATGTTCTGGCAGAAATTGCG

CGCGATCGCGGAAATGAAGAACTGCTTGAGAAAGCAGCGCGCCTTGCTGAGGAAGCAGCCCGCCAGGCGGAGGAGATTGCC
CGTCAGGCGCGCAAGGAAGGAAGCGCCCCGCCTCCCGTTCCTTCAACCCCCCCCACCCCAAGTCCGTCAGCTGGACAAGTAC
AACTTCAAGAAAGCGGCGGTGGGTTGGTTCAGGCGGGCGGCAGCCTTCGTTTGTCCTGTGCCGCCTCTGGCCGTACCTTTAGT
GAGTACGCAATGGGTTGGTTTCGCCAGGCCCCTGGTAAGGAACGTGAATTTGTTGCAACTATCTCATGGAGTGGTGGCTCTACT
TATTACACCCGTTCAGTAAAAGGACGTTTCACTATCTCCCGCGACAACGCGAAAAACACCGTCTACTTGCAAATGAATAGCCTG
AAGCCCGACGATACAGCAGTATATTATTGCGCGGCAGCGGGGTTAGGAACAGTAGTATCAGAGTGGGATTACGACTACGACTA
TTGGGGACAGGGCACTCAAGTAACAGTGTCAAGCGGACATCATCATCACCACCAC

**MGNLELALKALQILVNAAYVLAEIARDRGNEELLEKAARLAEEAARQAEEIARQARKEGNLELALKALQILVNAAYVLAEIARDRGN
EELLEKAARLAEEAARQAEEIARQARKEGNLELALKALQILVNAAYVLAEIARDRGNEELLEKAARLAEEAARQAEEIARQARKEG**
SAPPPVPSTPPTPSPSA<u>**GQVQLQESGGGLVQAGGSLRLSCAASGRTFSEYAMGWFRQAPGKEREFVATISWSGGSTYYTRSVKG
RFTISRDNAKNTVYLQMNSLKPDDTAVYYCAAAGLGTVVSEWDYDYDYWGQGTQVTVSS**</u>**GHHHHHH**

Table S2. Binding of trimeric tcTRP Anti-SARS CoV2 VHH domains to SARS RBD

CONSTRUCT	[a] Yield (mg/L)	[b] IC50 (nM)	[c] EC50 (nM)	[d] K_D (nM)	[d] k_a (1/nMs)	[d] k_{dis} (1/s)
**$tcTRP9x_3\_VHH678_3$**	15.0	1.64 (95% CI= 1.43 - 1.82)	2.06 (95% CI= 1.79 -2.34)	1.90 ± 0.03	8.23E+04 ± 1.00E+03	1.56E-04 ± 1.78E-06
**$tcTRP9x_3\_VHH679_3$**	17.5	1.41 (95% CI= 0.85 - 1.82)	5.52 (95% CI= 4.86 - 6.27)	12.1 ± 0.41	1.30E+04 ± 1.82E+02	1.57E-04 ± 4.83E-06
**$tcTRP9x_3\_VHH680_3$**	22.5	5.54 (95% CI= 4.39 - 6.97)	8.01 (95% CI= 6.94 - 9.28)	3.06 ± 0.06	1.12E+05 ± 1.93E+02	3.41E-04 ± 3.58E-06
**$tcTRP9x_3\_VHH681_3$**	15.0	0.78 (95% CI= 0.41 - 1.19)	0.64 (95% CI= 0.51 - 0.78)	0.43 ± 0.01	9.40E+04 ± 7.99E+02	3.98E-05 ± 3.36E-06
**$tcTRP9x_3\_VHH682_3$**	77.5	3.11 (95% CI= 2.47 - 3.70)	4.65 (95% CI= 4.21 - 5.13)	0.99 ± 0.04	1.11E+05 ± 2.32E+03	1.10E-04 ± 4.17E-06
**$tcTRP9x_3\_VHH683_3$**	40.0	2.57, (95% CI= 2.32 - 2.80)	2.69 (95% CI= 2.33 - 3.10)	1.21 ± 0.05	7.49E+04 ± 1.86E+03	9.05E-05 ± 3.32E-06
**$tcTRP9x_3\_VHH684_3$**	32.5	2.1 (95% CI= 1.58 - 2.48)	1.89 (95% CI= 1.59 - 2.25)	8.37 ± 0.14	3.99E+04 ± 2.19E+02	3.34E-04 ± 5.43E-06
**$tcTRP9x_3\_VHH685_3$**	32.5	1.02 (95% CI= 0.72 - 1.29)	1.88 (95% CI= 1.58 - 2.23)	1.40 ± 0.02	1.14E+05 ± 3.96E+02	1.59E-04 ± 1.68E-06

See **Figure 8** and **Supplemental Figure S5** for data related to the contents of this table.

Homotrimeric $tcTRP9x_3$-VHH_3 constructs are expressed in *E. coli*.

[a] Expression yields are presented in mg/L of culture.

[b] IC50 represents VHH concentration to achieve 50 % inhibition of infection of 293T-Ace2 cells by SARS-CoV-2 spike pseudotyped lentiviruses. The 95% confidence interval indicates the goodness of fit of the non-linear regression. Data is from a single experiment.

[c] ELISA EC50 denotes the VHH concentration required to achieve 50 % binding to SARS-CoV-2 RBD. The data we present are from a single ELISA experiment with experimental replicates. The 95% confidence interval given indicates the goodness of fit of the non-linear regression.

[d] Binding kinetics were measured using Biolayer interferometry (BLI). The rate of association and dissociation are calculated from concentration-dependent responses. The equilibrium dissociation constant (K_D) is given as a function of the rates of association and dissociation. Reported errors represent one standard deviation of the mean.

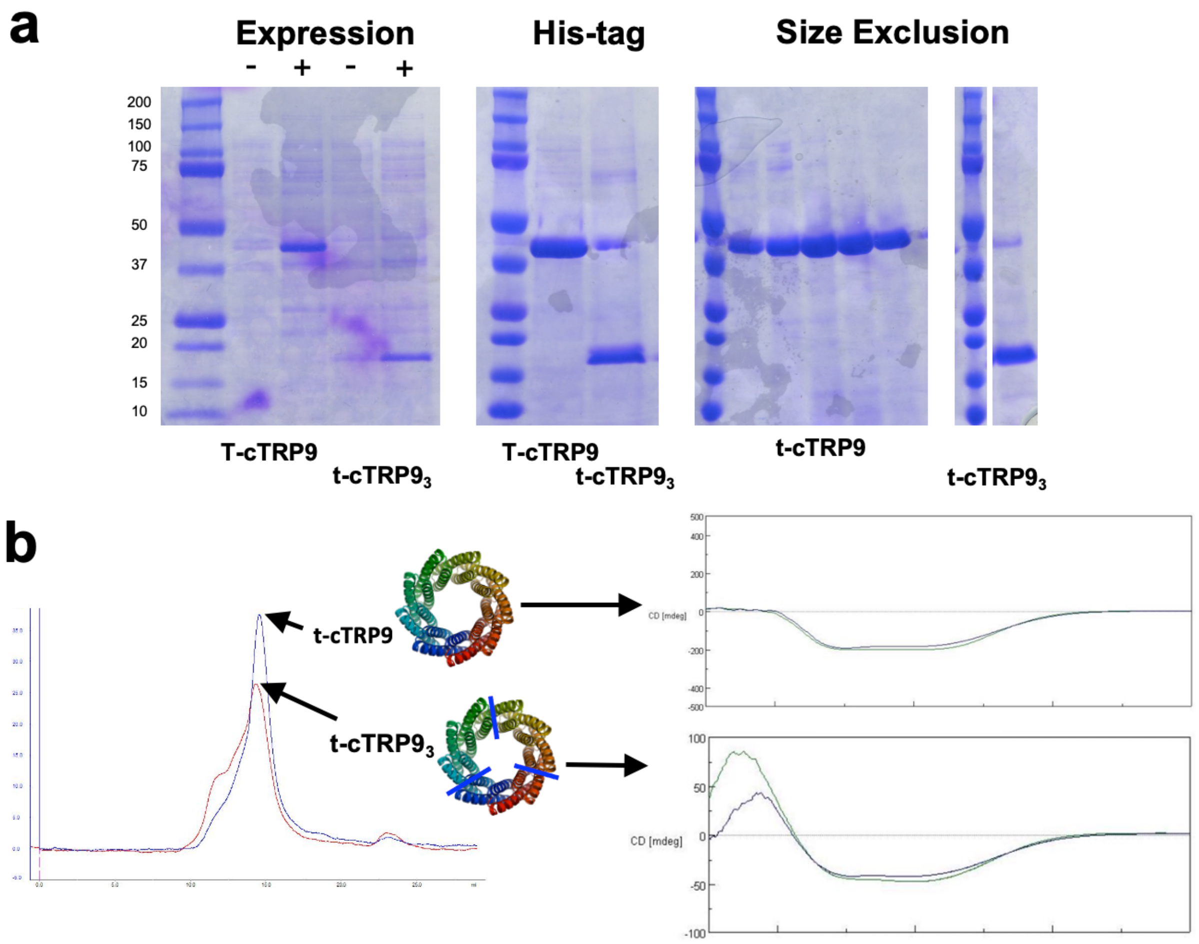

Supplemental Figure S1 (**Related to Figures 1, 3 and 4). Expression and purification of tcTRP9 and tcTRP9$_3$.** ***Panel a***: Induction and expression of each construct (left gel) was followed by affinity chromatography via an N-terminal polyhistidine tag (middle gel) and then size exclusion chromatography ('SEC'; right gels). ***Panel b:*** SEC elution of tcTRP9 and tcTRP9$_3$. Both constructs elute at approximately the same retention volume; the trimeric construct (tcTRP9$_3$) elutes slightly faster and with a preceding shoulder that may reflect dynamic sampling of a transient 'open' or partially dissociated complex with a larger average hydrodynamic radius during the column run. Right: Circular Dichroism (CD) spectra of the two constructs collected at 22° C and 95°C are extremely similar, indicating retention of structure at the higher temperature.

a

b

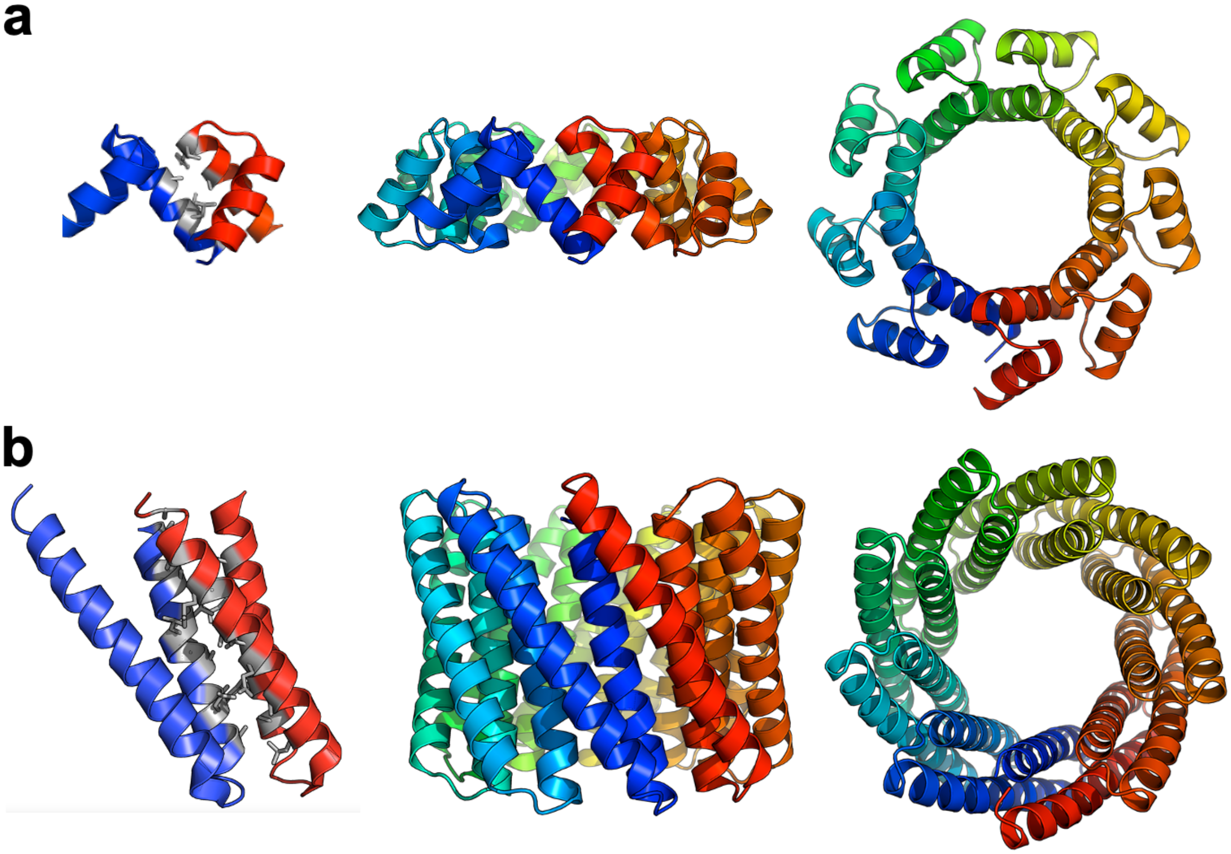

Supplemental Figure S2 (related to Figures 1, 3 and 4). Comparison of the dimensions and topology of a 9 repeat 'cTRP' (circular Tandem Repeat Protein as described in[13]) compared to a comparable 9 repeat 'tcTRP' (thick circular Tandem Repeat Protein) described in this study. Both structures are colored from blue at the N-terminal first repeat to red at the C-terminal last (ninth) repeat. The individual repeats have been converted from left-handed two-helix bundles (with each helix composed of 4 turns) to longer right-handed two-helix bundles (with each helix composed of 8 turns).

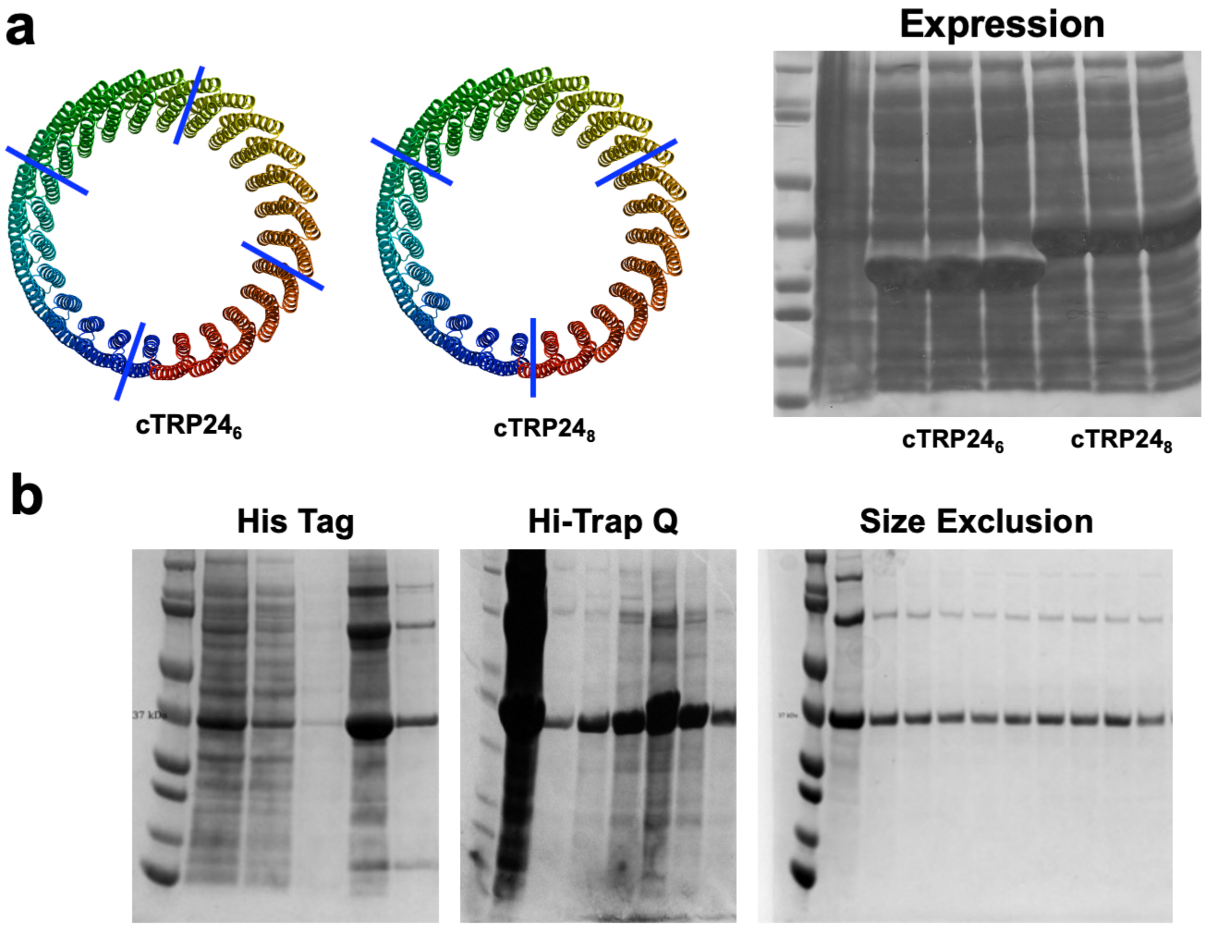

Supplemental Figure S3 (related to Figure 5). Expression of $tcTRP24_6$ and $tcTRP24_8$ and purification of $tcTRP24_8$. ***Panel a***: Induction of both constructs, which are each illustrated to the right. Both constructs are designed to form an assemblage corresponding to a tcTRP containing 24 total repeats; the first ($tcTRP24_6$) via tetramerization and the second ($tcTRP24_8$) via trimerization. ***Panel b:*** Purification of $tcTRP24_8$. The protein was purified in a three-step process corresponding to affinity chromatography via an N-terminal polyhistidine tag (left gel), ion exchange chromatography over a Hi-TrapQ column (middle gel) then size exclusion chromatography ('SEC'; right gel). The upper band on the fractions corresponding to the SEC elution corresponds to persistent folded protein after SDS-PAGE analysis; this behavior is often seen for highly thermostable proteins designed using the *RosettaDesign* software package.

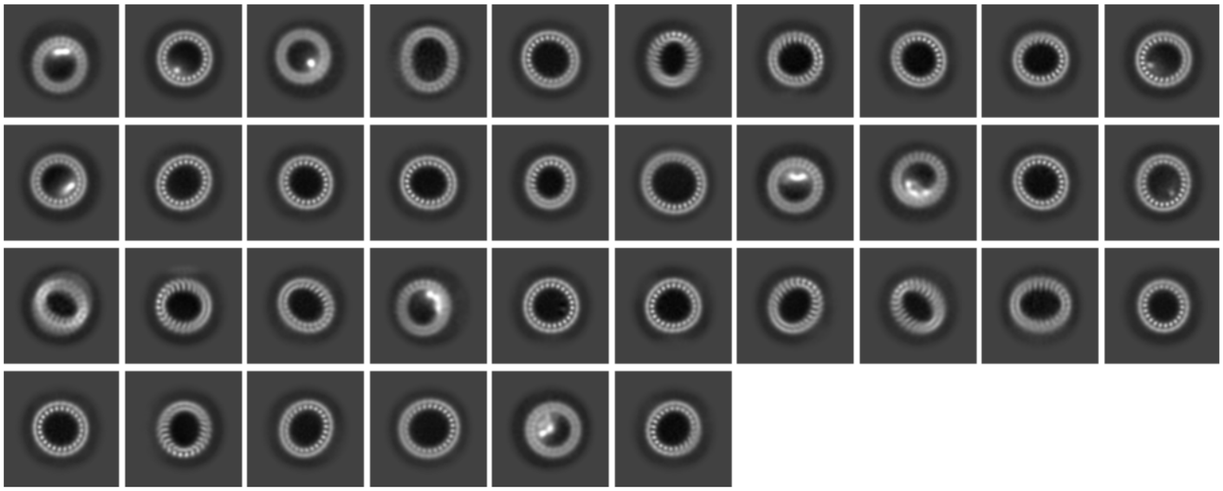

Supplemental Figure S4 (Related to Figure 6). 2-D projections of tcTRP24$_8$SS used for CryoEM reconstruction (Figure 5) validates the overall design and dimensions of that construct.

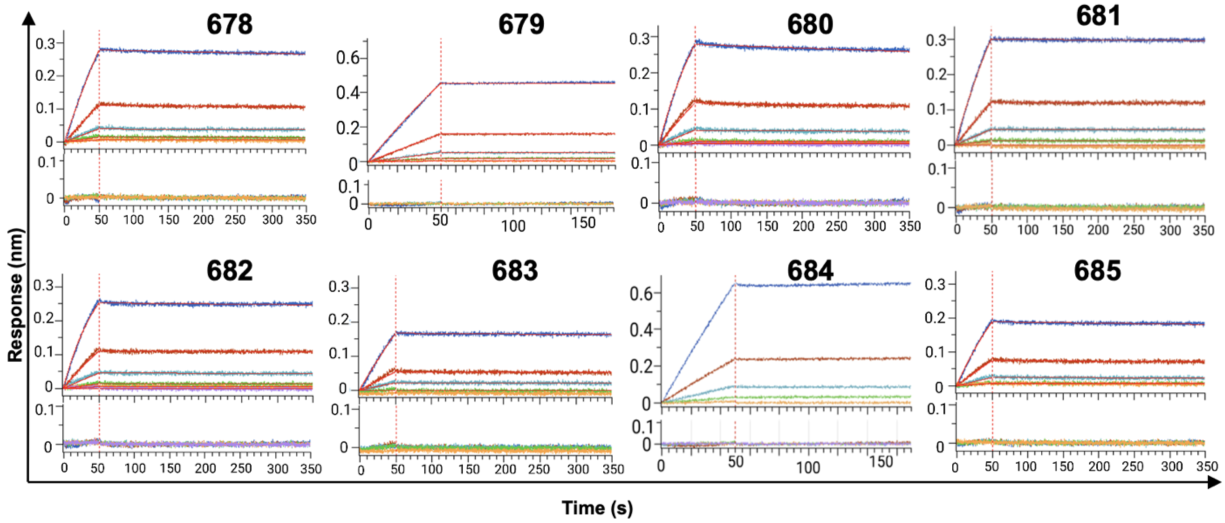

Supplemental Figure S5 (Related to Figure 8 and Supplemental Table S2). Representative binding sensorgrams for determination of kinetics of binding interactions and equilibrium dissociation constants (K_D) for the interaction between homotrimeric tcTRP9$_3$-VHH$_3$ constructs and SARS-CoV2 RBD.

BLI sensorgrams of the eight trimeric versions of SARS-CoV-2 targeting VHH, VHH 72, recorded at pH 7.2 were assayed for binding to the SARS CoV2 spike receptor binding domain (RBD) immobilized on a streptavidin biosensor. The apparent equilibrium binding constant (K_D) for each construct are determined from the rate of association (k_a), and rate of dissociation (k_{dis}) using the protocol described in the methods section. The rate of k_a and k_d are calculated from concentration-dependent responses. Values of K_D, k_a and k_d are indicated in **Supplemental Table S2**; dissociation constants ranged from 0.4 nM (construct 681) to 8.4 nM (construct 684). Kinetic analysis was performed using the HT 11.1.1.39 Data Analysis module (ForteBio). Results were double referenced. Both association and dissociation steps were used in 1:1 binding model Global data fitting model. The response sensorgrams for association and dissociation phases of the analysis and corresponding fit are given for each interaction. The residuals of the fits are plotted below the respective sensorgrams.
