# Supplementary material for: Design of functionalised circular tandem repeat proteins with longer repeat topologies and enhanced subunit contact surfaces
Source: Commun Biol. 2021 Oct 29;4:1240. doi: 10.1038/s42003-021-02766-y (PMC8556268; doi:10.1038/s42003-021-02766-y)
Supplement: Supplementary file 2 — Reporting Summary [file 42003_2021_2766_MOESM2_ESM.pdf]

nature research

Corresponding author(s): Stoddard, Barry L.

Last updated by author(s): 06-October-2021

Reporting Summary

Nature Research wishes to improve the reproducibility of the work that we publish. This form provides structure for consistency and transparency in reporting. For further information on Nature Research policies, see our Editorial Policies and the Editorial Policy Checklist.

Statistics

For all statistical analyses, confirm that the following items are present in the figure legend, table legend, main text, or Methods section.

n/a	Confirmed	
☐	☒	The exact sample size (n) for each experimental group/condition, given as a discrete number and unit of measurement
☐	☒	A statement on whether measurements were taken from distinct samples or whether the same sample was measured repeatedly
☐	☒	The statistical test(s) used AND whether they are one- or two-sided<br>*Only common tests should be described solely by name; describe more complex techniques in the Methods section.*
☒	☐	A description of all covariates tested
☒	☐	A description of any assumptions or corrections, such as tests of normality and adjustment for multiple comparisons
☐	☒	A full description of the statistical parameters including central tendency (e.g. means) or other basic estimates (e.g. regression coefficient) AND variation (e.g. standard deviation) or associated estimates of uncertainty (e.g. confidence intervals)
☒	☐	For null hypothesis testing, the test statistic (e.g. F, t, r) with confidence intervals, effect sizes, degrees of freedom and P value noted<br>*Give P values as exact values whenever suitable.*
☒	☐	For Bayesian analysis, information on the choice of priors and Markov chain Monte Carlo settings
☒	☐	For hierarchical and complex designs, identification of the appropriate level for tests and full reporting of outcomes
☒	☐	Estimates of effect sizes (e.g. Cohen's d, Pearson's r), indicating how they were calculated

Our web collection on statistics for biologists contains articles on many of the points above.

Software and code

Policy information about availability of computer code

Data collection	The computational design approach corresponded to a geometry-guided repeat computational strategy implemented in the Rosetta package with additional de novo design elements. Citations and additional details provided in Methods; program is publically available.<br><br>The computational approach used for structure determinations is also provided and cited in Methods; all programs are commonly used publically available software packages.
Data analysis	N/A

For manuscripts utilizing custom algorithms or software that are central to the research but not yet described in published literature, software must be made available to editors and reviewers. We strongly encourage code deposition in a community repository (e.g. GitHub). See the Nature Research guidelines for submitting code & software for further information.

Data

Policy information about availability of data

All manuscripts must include a data availability statement. This statement should provide the following information, where applicable:

- Accession codes, unique identifiers, or web links for publicly available datasets
- A list of figures that have associated raw data
- A description of any restrictions on data availability

The structures reported in this study have been deposited in the protein data base (www.rcsb.org) under PDB ID (entries 6XR1, 6XR2 and 7RDR) and the EMDB (entry 24425) and are available for immediate download. The original source data corresponding to all biochemical analyses have been added to the Harvard Dataverse depository (at https://dataverse.harvard.edu/dataverse/tcTRP) for public download and are also archived at our respective institutions and available upon request. Plasmids corresponding to tcTRP9 and tcTRP24 constructs described in this paper are available from the authors upon request and have also been provided to the Addgene public depository (IDs 175793 through 175800). Any remaining information can be obtained from the corresponding author upon reasonable request.

Field-specific reporting

Please select the one below that is the best fit for your research. If you are not sure, read the appropriate sections before making your selection.

[x] Life sciences [ ] Behavioural & social sciences [ ] Ecological, evolutionary & environmental sciences

For a reference copy of the document with all sections, see nature.com/documents/nr-reporting-summary-flat.pdf

Life sciences study design

All studies must disclose on these points even when the disclosure is negative.

Sample size	For all biochemical experiments (binding assays and pseudoviral neutralization assays) n = 3 independent replicates.
Data exclusions	No data exclusions.
Replication	All experiments were conducted in triplicate using independent aliquots of each protein. Data shown as the mean and standard deviation for n=3 measurements.
Randomization	N/A
Blinding	N/A

Reporting for specific materials, systems and methods

We require information from authors about some types of materials, experimental systems and methods used in many studies. Here, indicate whether each material, system or method listed is relevant to your study. If you are not sure if a list item applies to your research, read the appropriate section before selecting a response.

Materials & experimental systems

n/a	Involved in the study
[x]	[ ] Antibodies
[x]	[ ] Eukaryotic cell lines
[x]	[ ] Palaeontology and archaeology
[x]	[ ] Animals and other organisms
[x]	[ ] Human research participants
[x]	[ ] Clinical data
[x]	[ ] Dual use research of concern

Methods

n/a	Involved in the study
[x]	[ ] ChIP-seq
[x]	[ ] Flow cytometry
[x]	[ ] MRI-based neuroimaging
